# Antibiotic Administration Routes and Oral Exposure to Antibiotic Resistant Bacteria as Key Drivers for Gut Microbiota Disruption and Resistome in Poultry

**DOI:** 10.3389/fmicb.2020.01319

**Published:** 2020-07-07

**Authors:** Yang Zhou, Yu Li, Lu Zhang, Zuowei Wu, Ying Huang, He Yan, Jiang Zhong, Li-Ju Wang, Hafiz M. Abdullah, Hua H. Wang

**Affiliations:** ^1^Department of Food Science and Technology, The Ohio State University, Columbus, OH, United States; ^2^Department of Microbiology, School of Life Sciences, Fudan University, Shanghai, China; ^3^College of Food Science and Engineering of Technology, Guangzhou, China; ^4^College of Veterinary Preventive Medicine, Iowa State University, Ames, IA, United States; ^5^College of Food Science, Fujian Agriculture and Forestry University, Fuzhou, China; ^6^Department of Animal Science Poultry Facility, OARDC, Wooster, OH, United States; ^7^Department of Microbiology, The Ohio State University, Columbus, OH, United States

**Keywords:** antibiotic, administration routes, oral, injection, poultry, resistome, gut microbiota, opportunistic pathogens

## Abstract

Previous studies have identified oral administration of antibiotics and gut-impacting drugs as critical drivers for fecal antibiotic resistance (AR) and microbiome disruption in lab mice, but the practical implications of these findings have yet to be validated in hosts nurtured in conventional environment. Using ampicillin (Amp) as a way to extrapolate the general effect of antibiotics, this project examined the impact of drug administration routes on fecal microbiota and resistome using poultry raised in a teaching farm. AR genes were found to be abundant in the feces of young Leghorn chicks without previous antibiotic treatment. In chickens seeded with *bla*_CMY–2_^+^ *Escherichia coli*, 300 mg/kg body weight of Amp was orally administered for 5 days. This led to the fecal microbiota switching from Firmicutes occupied (95.60 ± 2.62%) and *Lactobacillus* rich, to being dominated by Proteobacteria (70.91 ± 28.93%), especially *Escherichia/Shigella*. However, when Amp was given via muscle injection, Firmicutes was mostly retained (i.e., from 83.6 ± 24.4% pre- to 90.4 ± 15.2% post-treatment). In control chickens without seeding with *bla*_CMY–2_^+^ *E. coli*, oral Amp also led to the increase of Proteobacteria, dominated by *Klebsiella* and *Escherichia*/*Shigella*, and a reduction of Firmicutes. Specifically within Firmicutes, *Enterococcus, Clostridium*, etc. were enriched but *Lactobacillus* was diminished. The fecal resistome including Amp^r^ genes was more abundant in chickens receiving oral Amp than those treated with muscle injection, but the difference was primarily within 1 log. The data illustrated that both drug administration routes and pre-existing gut microbiota have profound impacts on gut microbiome disruption when antibiotic treatment is given. In hosts nurtured in a conventional environment, drug administration route has the most evident impact on gut microbiota rather than the size of the targeted *bla*_CMY–2_^+^ gene pool, likely due to the pre-existing bacteria that are (i) less susceptible to Amp, and/or (ii) with Amp^r^- or multidrug resistance-encoding genes other than *bla*_CMY–2_^+^. These results demonstrated the critical interplay among drug administration routes, microbiota seeded through the gastrointestinal tract, AR, gut microbiota disruption, and the rise of common opportunistic pathogens in hosts. The potential implications in human and animal health are discussed.

## Introduction

The rapid rise of antibiotic resistance (AR) has raised serious public health concerns and led to the enforcement of policies to limit the uses of antibiotics in food animal production as well as human medicine. The evolution and enrichment of AR associated with antibiotic applications in concentrated food animal production operations is undeniable (Yurack, 1964; Levy et al., 1976; Levy, 1978; Founou et al., 2016). However, without prompt antimicrobial intervention, disease spread among animals can lead to severe losses in production, as well as more costly containment efforts (Casewell et al., 2003; McDevitt et al., 2006). So, how can these two issues be reconciled?

In the past decades, substantial studies have illustrated that multiple risk factors have contributed to AR development, enrichment, dissemination, and persistence. Therefore, targeted mitigation has become essential and deliverable. For instance, a large AR gene pool associated with foodborne microbiota was detected in ready-to-eat foods, redefining food consumption as a key avenue for disseminating AR bacteria and encoding genes to the general public. This discovery enabled successful mitigation of the largest foodborne AR gene pool associated with fermented dairy products in a few years, once multiple problematic starter and probiotic strains were removed from the product lines (Wang et al., 2006; Manuzon et al., 2007; Wang, 2010; Li et al., 2011). AR bacteria and AR genes are abundant and persistent in various hosts and environments, from wild animals, newborn babies never exposed to antibiotics, to food animals from organic production, and even EU farms that abandoned growth promotional antibiotics years ago (Borgen et al., 2000; Casewell et al., 2003; Johnsen et al., 2005; Sorum et al., 2006; Moritz and Hergenrother, 2007; Allen et al., 2010; Zhang et al., 2011). Multiple molecular mechanisms have been identified as contributors to AR persistent and niche fitness, even in the absence of antibiotic selective pressure (Andersson and Hughes, 2011; Harms et al., 2016; Singh et al., 2018; Bakkeren et al., 2019). Although eliminating AR bacteria associated with fresh produce and food animal products has been difficult to achieve so far at the production level, food processing treatments intended to inactivate pathogens are also effective against AR bacteria associated with these products (Wang, 2010). Nevertheless, the AR bacteria-rich feces released daily by billions of human and animals represent the most significant avenue impacting the pool of environmental AR bacteria and AR genes, subsequently spreading them to the global ecosystem and into food and hosts (Chee-Sanford et al., 2001; Nandi et al., 2004; Salyers et al., 2004; Doyle et al., 2006). Thus, there is an urgent need to identify key risk factors for fecal AR proliferation.

Using a lab mouse model and two antibiotics with differing pharmacological properties, Zhang et al. (2013) revealed that (i) oral administration of antibiotics, not the use of the antibiotic itself, is the direct cause of AR proliferation in feces and gut microbiota disruption, (ii) drug pharmacological properties also impact outcomes; for instance, for drugs at least partially excreted through the bile/fecal route instead of through the kidney/urine route, injection alleviates side effects, but does not eliminate them, and (iii) without oral seeding of AR bacteria, the targeted AR gene pools were not observed in feces even after 5 days of antibiotic treatment, regardless of administration route. Results from this study provided support for an alternative interpretation, that the rising trends of AR and chronic host health conditions associated with gut microbiota disruption could be largely due to oral administration of antibiotics. The potential to separate antibiotic applications from common detrimental side effects is encouraging, enabling effective drug intervention for disease prevention and treatment without fueling secondary problems. However, the results from lab animal studies need to be further validated in conventional settings.

Poultry represents the largest sector of the global food-producing animal industry. It is one of the largest consumers of antibiotics, as well as a key producer of animal waste with significant environmental impact. According to the FDA, majority of the antibiotics used in poultry production are administrated orally, mixed into feed or water (U.S. Food and Drug Administration, 2018, 2019). AR bacteria are abundant across the poultry production chain worldwide, regardless of direct exposure status to antibiotics (Baron et al., 2018; Daehre et al., 2018b; Apostolakos et al., 2019). Moreover, birds and mammals have distinctive anatomy and physiology, and zoonotic pathogens have the ability to directly impact human health, including those originating in poultry (Knudsen et al., 2018; Borges et al., 2019). Therefore, this study examined the impact of antibiotic administration routes and oral exposure to AR bacteria in a poultry production environment free of growth-promotional uses of antibiotics. This was done in order to assess the applicability of the previous findings in conventional settings and a diversified set of animal hosts, as well as to explore the potential implications for human and animal health.

## Materials and Methods

### Isolation and Identification of *bla*_CMY–2_^+^ Strains and Culture Preparation for Inoculation

Poultry fecal isolates were retrieved from Columbia Blood Agar plates and cultivated separately in Columbia Broth (Becton, 100 Dickinson and Company, Franklin Lakes, NJ, United States) at 37°C. Three *bla*_CMY–2_^+^ strains used in this study were isolated from the feces of two 5-day-old broiler chickens without antibiotic exposure and confirmed to be *E. coli* by 16S rDNA sequence analysis. The key features of the strains are summarized in Table 1.

**TABLE 1:** The *bla*_CMY–2_^+^ *E. coli* strains used in the study.

Cocktail Strain ID	Resistance	MIC^1^ (μ g/mL)	AR gene^1^	DGGE^2^ cluster
*Escherichia coli* CA-1	Amp^r^ Axo^r^ Rif^r^	512 <8 4	*bla*_CMY–2_	1
*Escherichia coli* CA-4	Amp^r^ Axo^r^ Rif^r^	512 <8 >4	*bla*_CMY–2_	1
*Escherichia coli* CA-20	Amp^r^	512	*bla*_CMY–2_	1
	Axo^r^	16		
	Rif^r^	4		

*Amp, ampicillin; Axo, ceftriaxone; Rif, rifampin. ^1^MIC and PCR screening for AR genes were conducted following published procedures (Wang et al., 2006). ^2^DGGE assessment was detailed in section “Materials and Methods.”*

To prepare for chicken inoculation, cells from 1 mL of overnight culture of each strain were collected by centrifugation (8000 × *g*, 1 min), washed once, and re-suspended in 1 mL saline. The final inoculation cocktail contained 10^6^ CFU/mL *E. coli* cells from three strains mixed at 1:1:1 ratio and was used to seed the chicken gut by gavage feeding.

### Feed Treatment and Quality Measurement

Standard chicken diet P10109 prepared by Ohio Agricultural Research and Development Center (OARDC) Poultry Facility was used in the study. Feed composition was illustrated in Supplementary Table S1. Poultry feed, two pounds each in autoclaving-safe box (30 cm × 30 cm × 10 cm) or cylinder jar (20 cm in diameter, 20 cm in height), was processed in a sterilizer (AMSCO Renaissance series 3021, Mentor, OH, United States) under gravity mode at 121°C, 103.4 kPa for 15 min, cooled down to room temperature, and heated with the same parameters again to minimize bacterial population including spore-forming cells. The bacterial population of the resulting feed was less than 5 × 10^2^ CFU/g, assessed by plate counting on Plate Count Agar (Becton, 100 Dickinson and Company, Franklin Lakes, NJ, United States) supplemented with 100 μg/mL cycloheximide (Sigma-Aldrich) and Columbia Blood Agar base (CBA, Becton Dickinson and Company, Franklin Lakes, NJ, United States), supplemented with 5% defibrinated sheep blood (Fisher Scientific, Hampton, NH, United States) and 100 μg/mL cycloheximide (Sigma-Aldrich).

### Animals, Experimental Design, and Antibiotic Administration

The experiment was conducted following animal protocol No. 2012A00000061, approved by the Institutional Animal Care and Use Committee, The Ohio State University, Columbus, OH, United States. To assess the baseline resistome in newly hatched chickens, 5 Leghorn chickens hatched within one week were purchased from a local breeder, and another 10 chickens from two batches were hatched at the OARDC Poultry Research Farm. To evaluate the impact of antibiotic administration routes, Leghorn chickens used in the controlled experiments were hatched and maintained at the OARDC Poultry Research Farm (295 chickens) or maintained at the OARDC Turkey Research Center (30 chickens).

Ampicillin (Amp) was chosen in the study because of its application history in the poultry industry, both in the U.S. and worldwide, and also because the abundance of the targeted gene was still low enough at baseline, for changes to be detected in the study, as opposed to some of the other commonly used antibiotics such as tetracycline. The range of application dosage is quite wide, ranging from 20 to 400 mg/kg body weight being used in poultry production and research (Donoghue et al., 1996; Commission of Chinese Veterinary Pharmacopoeia, 2010; Zhao et al., 2015). Thus, a dosage of 300 mg/kg was chosen in this study.

Chickens were randomly distributed into eight groups (Table 2). Supplementary Figure S1 illustrates the experimental flow for each round of the experiment. A total of 325 chickens (two birds per cage with separate feed and water supply, controlled temperature, filtered air in the room, and heat-treated feed and distilled water) were used in multiple rounds of the study. Chickens receiving intramuscular (IM) or oral (per os, PO) ampicillin administration after *bla*_CMY–2_^+^ strains inoculation were designated as Amp-IM and Amp-PO. Chickens receiving IM- or PO-ampicillin administration without prior inoculation of the *bla*_CMY–2_^+^ *E. coli* strains were designated as NI-Amp-IM and NI-Amp-PO. Chickens received IM- or PO-saline after the *bla*_CMY–2_^+^ *E. coli* inoculation were designated as Saline-IM and Saline-PO, which were collectively referred to as Sham group. Chicken groups received neither the *bla*_CMY–2_^+^ *E. coli* inoculation nor Amp but saline administration were defined as NI-Saline-IM and NI-Saline-PO. Both Amp-IM and Amp-PO experimental groups had at least 13 cages of chickens from 4 rounds of assessments. From Day 5 (D5) to Day 8 (D8) post-hatching, chicks were inoculated with the *bla*_CMY–2_^+^ *E. coli* cocktail (0.2 mL/bird, 10^6^ CFU/mL) every 24 hrs for 4 consecutive days via gavage feeding using 20 ga × 1.5 in an animal feeding needle (Fine Science Tools, Foster City, CA, United States). Controls were fed with 0.2 mL of saline during the inoculation period. Chicks were then reared in cages for 11 days until Day 20 (D20), allowing the microbiota to settle. Chickens in Amp-PO, Amp-IM, NI-Amp-PO, and NI-Amp-IM groups received antibiotic administration from D20. Antibiotics were administered via gavage feeding using 20 ga × 1.5 feeding needle or via breast intramuscular injection using 1 mL insulin syringe (Becton, Dickinson and Company, Franklin Lakes, NJ, United States). Chicks were administered with ampicillin or saline once a day for 5 consecutive days.

**TABLE 2:** Leghorn chicken groups subjected to marker cocktail inoculation and antibiotic administration treatments.

Group	AR carrier inocula	Antibiotic administration			
		PO	IM*	PO	IM*
			
		Ampicillin 300 mg/kg		Saline (control)	
Amp-PO	*E. coli* cocktail	+	–	–	–
Amp-IM	*E. coli* cocktail	–	+	–	–
Saline-PO	*E. coli* cocktail	–	–	+	–
Saline-IM	*E. coli* cocktail	–	–	–	+
NI-Amp-PO	–	+	–	–	–
NI-Amp-IM	–	–	+	–	–
NI-Saline-PO	–	–	–	+	–
NI-Saline-IM	–	–	–	–	+

Saline-PO and Saline-IM were collectively used as Sham group, while NI-Saline-PO and NI-Saline-IM were collectively used as Control groups in some analysis. *IM, intramuscular injection.

Fresh feces of each broiler chicken subject were collected on-site in the rearing facility, stored on ice, and transported to the lab within 4 h for microbial assessments. Fecal samples were collected once a week before antibiotic treatment, once a day during antibiotic administration, and once every three days after antibiotic withdrawal up to 14 days from initial antibiotic exposure. During antibiotic treatment, the daily fecal sample collection was carried out before drug administration practice.

### Quantification of Culturable Bacteria in Fecal Microbiota

Fresh fecal samples from 20 chickens randomly picked were subjected to culture recovery from D20. Fecal microbiota was recovered on Columbia Blood Agar base (CBA, Becton Dickinson and Company, Franklin Lakes, NJ, United States) supplemented with 5% defibrinated sheep blood (Fisher Scientific, Hampton, NH, United States) and 100 μg/mL cycloheximide (Sigma-Aldrich). CBA plates supplemented with 32 μg/mL of ampicillin sodium (Life Technologies, Grand Island, NY, United States) were used to recover Amp^r^ bacteria. The dry mass of chicken feces was spun down by centrifugation at 4°C at full speed (Eppendorf 5415R, Germany) and homogenized in stomacher bags by a stomacher (Seward Stomacher 80 Lab System, United Kingdom). Homogenized samples were serially diluted in sterile saline and plated on corresponding agar plates. The plates were incubated at 37°C for 48 h in a GasPak 150 anaerobic system with GasPak EZ anaerobe container system sachets (Becton Dickinson and Company). The upper and lower detection limits of the plate counting enumeration method are 10^10^ CFU/g and 10^2^ CFU/g, respectively.

### DNA Extraction

Total DNA were extracted from the dry mass of chicken feces. DNA extraction followed a published method for real-time quantitative PCR (qPCR) and denaturing gradient gel electrophoresis (DGGE) analyses (Yu and Morrison, 2004).

### Real-Time Quantitative PCR

TaqMan real-time PCR protocol was used to assess representative AR genes, and 16S rDNA gene pools in total DNA extracted from chicken feces as described previously (Zhang et al., 2013). The primers and probes for gene *bla*_CMY–2_, *tetL*, *tetM*, *tetS*, *sul1*, *sul2*, and 16S rDNA are listed in Table 3. The primers were synthesized by Sigma-Aldrich (St. Louis, MO, United States) and the probe was synthesized by Biosearch Technology Inc. (Novato, CA, United States). Each sample was assessed and analyzed in duplicates on a CFX96 system (Bio-Rad, Hercules, CA, United States).

**TABLE 3:** Primers and probes used in AR gene pool quantification.

Primer and probe	Sequence (5′-3′)	References
*bla*_CMY–2_ FP	GCCGTTGATGATCGAATC	Zhang et al., 2013
*bla*_CMY–2_ RP	GCGTATTGGCGATATGTAC	
*bla*_CMY–2_ probe	6FAM-AGTTCAGCATCTCCCAGCCTAATCC-BHQ1	
*tet*S FP	GTATGTTCATCTTTCTAAG	Li and Wang, 2010
*tet*S RP	GCAATAACATCTTTTCAAC	
*tet*S probe	6FAM-CCATGTGTCCAGGAGTATCTAC-BHQ1	
*tet*L FP	CGTCTCATTACCTGATATTGC	
*tet*L RP	AGGAGTAACCTTTTGATGCC	
*tet*L probe	6FAM-AACCACCTGCGAGTACAAACTGG-BHQ1	
*tet*M FP	GAACATCGTAGACACTCAATTG	
*tet*M RP	CAAACAGGTTCACCGG	
*tet*M probe	6FAM-CGGTGTATTCAAGAATATCGTAGTG-BHQ1	
*sul*1 FP	CACCTTCGACCCGAAG	Zhang, 2012 (Unpublished data)
*sul*1 RP	TTGAAGGTTCGACAGCACG	
*sul*1 probe	6FAM-TCGACGAGATTGTGCGGTTCTTCG-BHQ1	
*sul*2 FP	GATATTCGCGGTTTTCCAGA	
*sul*2 RP	CAAAGAACGCCGCAATGT	
*sul*2 probe	6FAM-ATCATCTGCCAAACTCGTCGTTATGC-BHQ1	
16s FP	TCCTACGGGAGGCAGCAGT	Nadkarni et al., 2002
16s RP	GGACTACCAGGGTATCTAATCCTGTT	
16s probe	6FAM-CGTATTACCGCGGCTGCTGGCAC-BHQ1	

*FP, forward primer; RP, reversed primer.*

For baseline AR gene quantification, fecal samples were collected on D5 from a total of 15 chickens from 3 batches, including 5 vendor-hatched chickens and 10 local-hatched chickens from 2 batches (5/batch). To examine the impact of antibiotic administration routes on the changes of the *bla*_CMY–2_ gene abundance, a total of 181 chickens were enrolled for real-time PCR analysis from 6 rounds of experiments. In each round of experiment, fecal samples of at least three randomly picked chickens from 3 different cages were subjected to real-time quantitative PCR analysis from each treatment group. The presented figures were constructed by data from at least five fecal samples from chickens in five different cages of each group.

### DGGE Analysis

While animals from different cages were used as independent unit for DGGE data presentation to avoid compounding error, in most cases both chickens from the same cage were assessed by DGGE to identify unusual outliers. A total of 111 chickens were assessed by DGGE. The experimental groups of Amp-PO and Amp-IM each had 19 chickens from 11 cages, the control groups each had at least 14 chickens from 7 cages. The 16S rDNA V3 region was used for amplification of partial 16S rDNA gene following a published procedure (Muyzer et al., 1993). The sequences of PCR primers used were 16S-357F-GC 5′-CGCCCGCCGCGCGCGGCGGGCGGGGCGGGGGCACGGG GGGCCTACGGGAGGCAGCAG-3′ and 16S-518R 5′-ATTACCGCGGCTGCTGG-3′; products were loaded on to an 8% acrylamide gel with a urea gradient from 40 to 60%. Electrophoresis was performed at 60°C, 83 V for 16 h using the Dcode system for DGGE (Bio-Rad, Hercules, CA, United States) (Muyzer et al., 1993). The finishing gel was stained with 0.01% ethidium bromide and imaged under ChemiDoc XRS system (Bio-Rad, Hercules, CA, United States). The dominant DNA band was recovered and sequenced.

### 16S rDNA Amplicon Sequencing and Shotgun Metagenomic Analysis

For 16S rDNA amplicon sequencing, a total of 82 chickens were enrolled. Except for the last round, individually sequenced samples were collected from chickens located in separated cages. Each pooled samples of the treatment and control groups consisted of feces from three additional chickens, which were also located in different cages. In the last round of experiment presented in Supplementary Figure S6, fecal samples all the chickens were subjected to individual sequencing. The V4/V5 region of the 16S rDNA gene were amplified following the standard protocol for 16S Metagenomic Sequencing Library Preparation (Illumina support, 2013), and the products were sequenced on Illumina Miseq (2 × 250 bp paired-end run) at OARDC Molecular and Cellular Image Center (individual samples). Paired-end reads joining and quality filtering were performed on Qiime2 following DADA2 procedure. Phylogenetic analysis and taxonomic assignments were conducted using Greengenes database (version 13_8). Diversity analysis was performed on the Qiime2 following standard procedure. Krona chart of the microbiota composition were generated from the sequences obtained from QIIME (Ondov et al., 2011).

For shotgun metagenomic analysis, total fecal DNAs, consisting of pooled samples of four chickens from two different cages per treatment or control group were sent to the Nationwide Children’s Hospital (Columbus, OH, United States) for sequencing quality control analysis, and sequenced on an Illumina HiSeq 2500 system (2 × 150 bp paired-end run) (Illumina Inc; San Diego, CA, United States). The raw reads were trimmed and quality-controlled using Trim Galore^1^ software with default parameters. Clean reads were processed using DeepARG platform and for AR gene quantification (Arango-Argoty et al., 2018).

### Statistics

Statistical analysis of the metagenomics data was based on the complete sample profile as expressed by the pattern of operational taxonomy units (OTUs) and the relative abundance (percentage) of individual OTU in each sample. For the relative abundance of bacterial population, the results were expressed as means ± standard error (SD). Inter-group comparisons were done with unpaired t tests (*Lactobacillaceae* abundance analysis) or Mann–Whitney *U* test (*Enterobacteriaceae* abundance analysis). Four-way comparisons (Amp-PO vs. Amp-IM vs. Sham vs. control) were done with Kruskal–Wallis test. The impacts of administration routes on the quantity dynamic of gene *bla*_CMY–2_ and 16S rDNA gene was analyzed with Linear Mix Model in SPSS (version 19.0). Significance was declared at *P* < 0.05.

## Results

### Antibiotic Resistant Bacteria and AR Gene Reservoir in Fecal Microbiota of Newly Hatched Poultry

**FIGURE 1:**
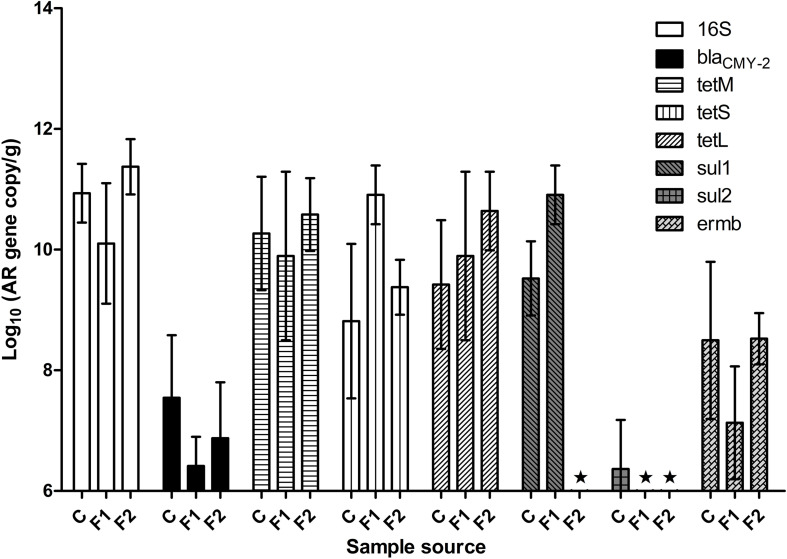
The abundance of representative AR genes and 16S rDNA in newly hatched chicken feces. The lowest detection limit was 10^6^ copies/g. C, fecal samples from vendor-hatched chickens. F1&F2, fecal samples from chickens hatched within facility from different batches. ★, Below detection limit.

Figure 1 illustrated fecal AR background by real-time PCR. The abundance of fecal 16S rDNA ranged between 10^10^ and 10^11^ copies/g feces. Representative AR genes *bla*_CMY–2_, *tetL, tetM*, *tetS*, and *ermB* were detected in the feces of all three batches of chickens. The three tetracycline-resistance (Tet^r^) genes were highly abundant across all the samples, followed by gene *ermB*. The baseline abundance of *bla*_CMY–2_ gene was relatively low. The abundance of *sul1* and *sul2* varied significantly among the three batches: *sul1* was highly abundant in vendor-hatched chickens and the first batch of OARDC facility-hatched chickens, but was below the detection limit in the second facility-hatched batch; the *sul2* gene was barely detected only in the vendor-hatched chickens. AR gene(s) with low baseline abundance were expected to respond to antibiotic intervention with detectable changes, making *bla*_CMY–2_ and *sal2* candidates as marker genes. While Amp can be delivered via drinking water, some sulfonamides have poor solubility in water. Thus, Amp was used in further antibiotic intervention assessments, and *bla*_CMY–2_ gene was chosen as the marker gene.

When fecal samples of 20 chickens hatched in facility were assessed for cultivable ampicillin-resistance (Amp^r^) bacterial population, an average of 8.0 ± 0.6 log CFU/g total cultivable bacteria were recovered on CBA plates, while Amp^r^ bacteria were detected in 42% of the examined chickens. This result confirmed that Amp^r^ bacteria naturally colonized in certain chickens.

### The Impact of Antibiotic Administration on *bla*_CMY–2_ Gene Pool

**FIGURE 2:**
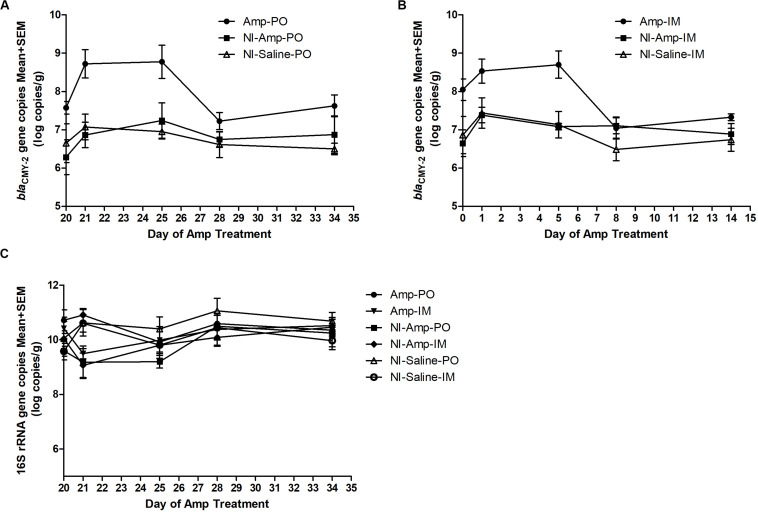
Real-time PCR quantification of fecal *bla*_CMY–2_ gene pool and 16S rDNA gene pool in Amp (300 mg/kg body weigh/day)-treated chickens. The change of *bla*_CMY–2_ gene pool in chicken fecal microbiome under Amp treatment by **(A)** oral administration and **(B)** muscle injection. **(C)** The change of 16S rDNA gene pool in chicken fecal microbiome. The detection limit of *bla*_CMY–2_ and 16S rDNA gene pools in this study is 5 log10 copies/g. The error bars represent standard deviations of the data from animal subjects used in the study. D24 was the last day of Amp administration.

The 16S rDNA gene pool of all treatment groups remained quite stable (Figure 2C), with abundance between 9 to 11 log10 copies/g of chicken feces. Compared to non-inoculated groups, inoculated groups (Amp-PO, Amp-IM) had larger *bla*_CMY–2_ gene pool before Amp treatment (D20), likely due to colonization of the inoculated *bla*_CMY–2_^+^ strains in chicken GI tracts (Figures 2A,B). Administration of 300 mg/kg of Amp led to a detectable increase of the *bla*_CMY–2_ gene pool in Amp-PO and Amp-IM groups, starting from the second day of antibiotic administration (D21), maintained during Amp administration period (to D24), and dropped after antibiotic withdrawal. But, despite the observation that *bla*_CMY–2_ gene pool size increased by 1.2 ± 0.7 log in Amp-PO group from D20 to D21 and by 0.5 ± 0.8 log in Amp-IM group, the difference between Amp-PO and Amp-IM treatment by real-time PCR was statistically insignificant (*P* = 0.054).

Without inoculating the *bla*_CMY–2_^+^ marker strains, however, the natural *bla*_CMY–2_^+^ gene pool in NI-Amp-PO and NI-Amp-IM groups did not have a detectable response to Amp administration, similar to the blank control groups NI-Saline-PO and NI-Saline-IM (*P* = 0.104).

### Impact of Antibiotic Administration Routes on Poultry Fecal Resistome

**FIGURE 3:**
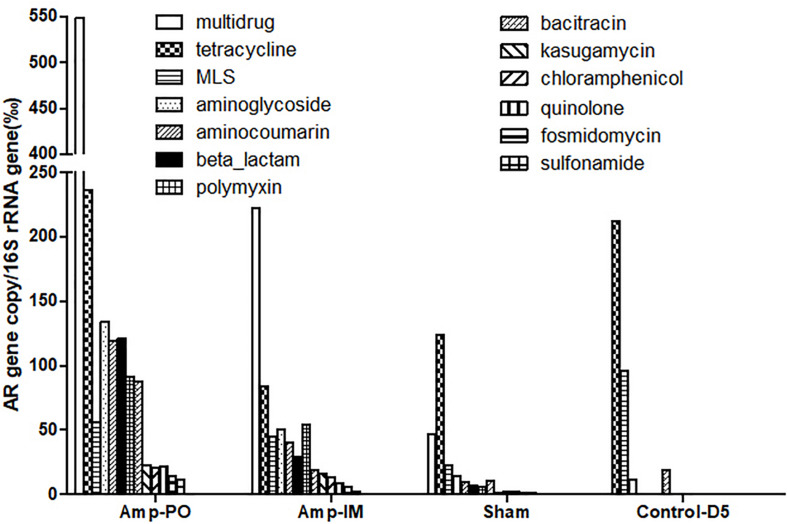
Abundance of AR genes in fecal microbiome of chickens after Amp or control treatments. Each bar represented the abundance of a group of AR genes against certain antibiotics.

Figure 3 illustrated the resistome by shotgun metagenomic sequencing. Consistent with the real-time PCR results in Figure 1, Tet^r^ genes were prevalent at early life of chickens as examined, and their abundance remained relatively high throughout the experimental period in all treatment groups. Administration of Amp, whether PO or IM, led to the increase of abundance of most AR genes in the fecal microbiota with a decrease of the Tet^r^ genes. Particularly, the increase of β-lactam and bacitracin-resistance genes were more than fourfold in Amp-PO than those in Amp-IM. Moreover, the rapid rise of multidrug-resistance genes was most evident in Amp-PO, which was more than 10- and 2-fold of those in Sham and Amp-IM, respectively.

The data indicated that switching the administration route from PO to IM had more substantial impact on the gut microbiota than simply the *bla*_CMY_ gene pool, resulting in significant changes in resistome. Further analysis of the β-lactam-resistance gene pool showed significant accumulation of *bla*_CMY_ and *bla*_AMP_ genes in the pooled Amp-PO sample. Particularly, the *bla*_CM__*Y*_ genes of Amp-PO had the most significant increase, reaching almost nine times that of Amp-IM group, and this gene family includes the *bla*_CMY–2_ gene carried by the *E. coli* marker strains (Supplementary Tables S2, S3).

### The Impact of Administration Routes on Poultry Fecal Microbiota

As illustrated in Figure 5 and Supplementary Figure S3 (D20 before Amptreatment), phylum Firmicutes dominated in normal chicken fecal microbiota, accounting for up to over 98% detected sequences (95.6 ± 2.6%), while the abundance of phylum Proteobacteria was primarily less than 5% of the total sequences. Without further antibiotic treatment, seeding *bla*_CMY–2_^+^ *E. coli* marker strains had undetectable or very limited impact on the abundance of Proteobacteria. Supplementary Figure S3 further illustrates that although the detailed compositions of fecal microbiota varied among individual subjects, without exception the phylum Firmicutes, and within it the family *Lactobacillaceae*, dominated poultry fecal microbiota.

**FIGURE 4:**
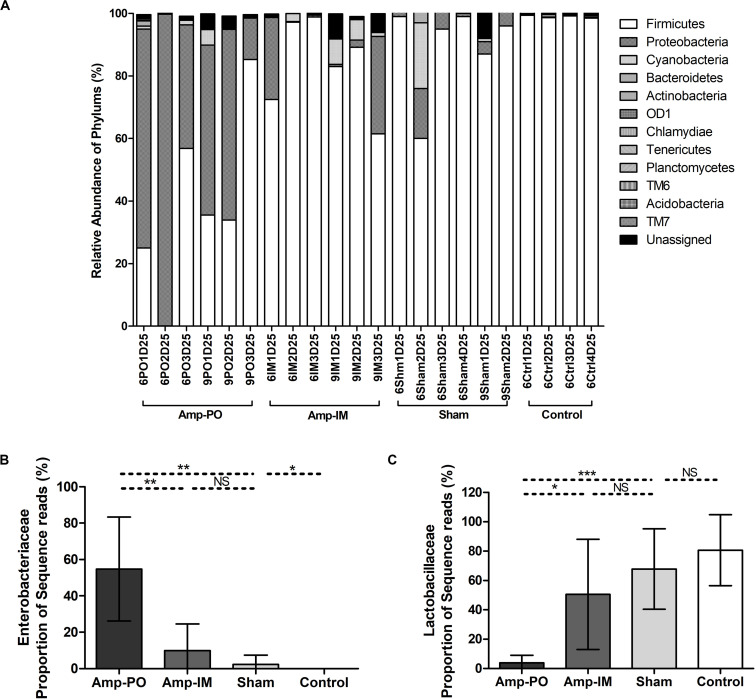
Impact of antibiotic administration route on the chicken fecal microbiota. **(A)** Overall bacterial profile plot of the detected phylum in fecal microbiota of chicken after antibiotic administration. **(B)** Impact of antibiotic administration route on the abundance of *Enterobacteriaceae* in chicken fecal microbiota. **(C)** Impact of antibiotic administration route on the abundance of *Lactobacillaceae* in chicken fecal microbiota. Sham: chicken inoculated with marker *bla*_CMY–2_^+^ *E. coli*, no Amp administration. Control: chicken without inoculation of marker *bla*_CMY–2_^+^ *E. coli*, no Amp administration. *p < 0.05; **p < 0.01; ***p < 0.001.

**FIGURE 5:**
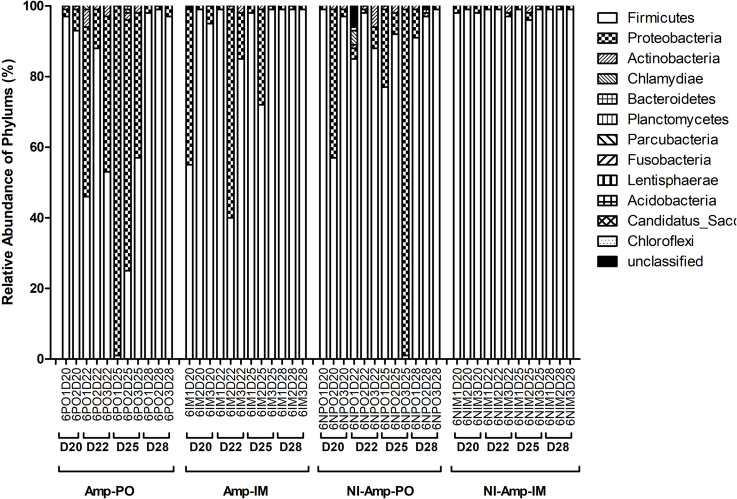
Dynamic change of microbial composition of chicken fecal microbiota during antibiotic treatment. Overall bacterial profile plot of the detected phylum in fecal microbiota of chicken in different treatment groups during antibiotic treatment.

Oral administration of Amp, however, significantly changed the profile of fecal microbiota, especially the Firmicutes/Proteobacteria ratio (Figures 4A, 5 and Supplementary Figures S4A,C). The abundance of phylum Proteobacteria overturned from mostly less than 1% to an average of over 50% among examined chickens, including as much as over 99% of the total population in one subject (Figure 4, Amp-PO and Supplementary Figure S4A, Proteobacteria 70.9 ± 28.9%). The lowest detected abundance of Proteobacteria was still over 40% of the population. On the other hand, Amp delivered by muscle injection had relatively modest impact on chicken gut microbiota (Figure 4A, Amp-IM; Supplementary Figure S4B). The average abundance of Proteobacteria in Amp-IM group was less than 32% and 4 out of 6 chicks had less than 5% relative abundance, similar to the Sham group without any ampicillin exposure. These results suggested that oral administration of antibiotic posed more substantial selective pressure on the GI microbiota compared to injection.

**FIGURE 6:**
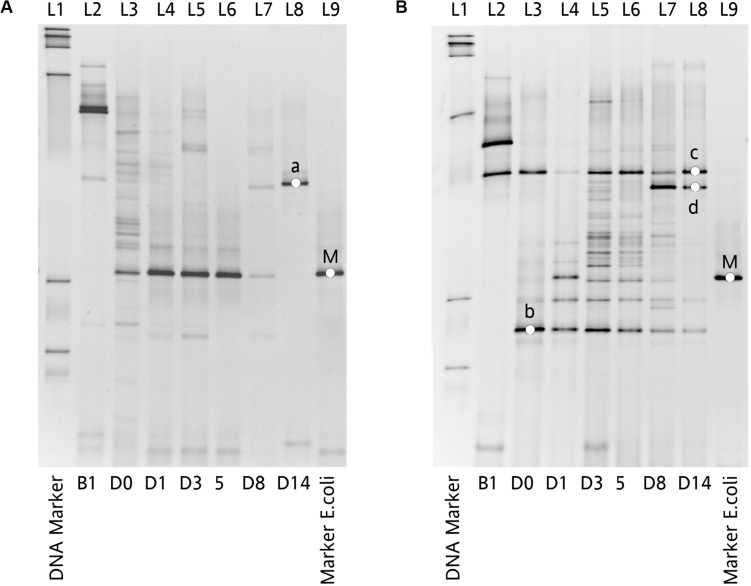
Impact of Amp treatment on dominant microbial profiles by DGGE assessment of 16S rDNA gene amplicons of total fecal DNA from inoculated chicken. **(A)** Microbial profiles of chicken fecal in Amp-PO, and **(B)** microbial profiles of chicken fecal in Amp-IM. Lane 1: 100 bp DNA ladder; Lane 2: before inoculation of marker strain; Lane 3: after inoculation but before Amp administration; Lane 4–6: 1st, 3rd, and 5th days with Amp exposure; Lane 7 and 8: 3rd and 9th days with Amp lifted; Lane 9: *bla*_CMY–2_^+^ *E. coli*. a: *Lactobacillus* sp.; b: *Lactobacillus* sp.; c: *Lactobacillus* sp.; d: *Lactobacillus* sp.; M: Inoculated *Escherichia coli*.

The dynamic of chicken fecal microbiota was tracked by 16S rDNA high-throughput sequencing throughout the antibiotic treatment (Figure 5). Firmicutes was the most abundant phylum before antibiotic treatment, consistent with previous data on normal chicken GI microbiota composition (Wei et al., 2013). With inoculation of Amp^r^ *E. coli*, the abundance of Proteobacteria in Amp-PO group increased substantially during Amp treatment, becoming the most dominant population in the fecal microbiota. However, the increase of Proteobacteria was modest in Amp-IM group and the dominance of Firmicutes was better preserved accordingly. DGGE analysis showed the similar dynamics of microbiota in Amp-PO and Amp-IM samples. Also illustrated by DGGE, oral Amp significantly induced amplification of the marker *E. coli* strains (M) in the chicken fecal microbiota (Figure 6A). Five days of oral feeding of Amp led to the dominance of *E. coli* and reduction of other bacterial subpopulations. However, the profiles of dominant fecal microbiota in chickens retained their diversity during Amp treatment by muscle injection (Figure 6B), indicating a milder selective pressure in GI tract.

**FIGURE 7:**
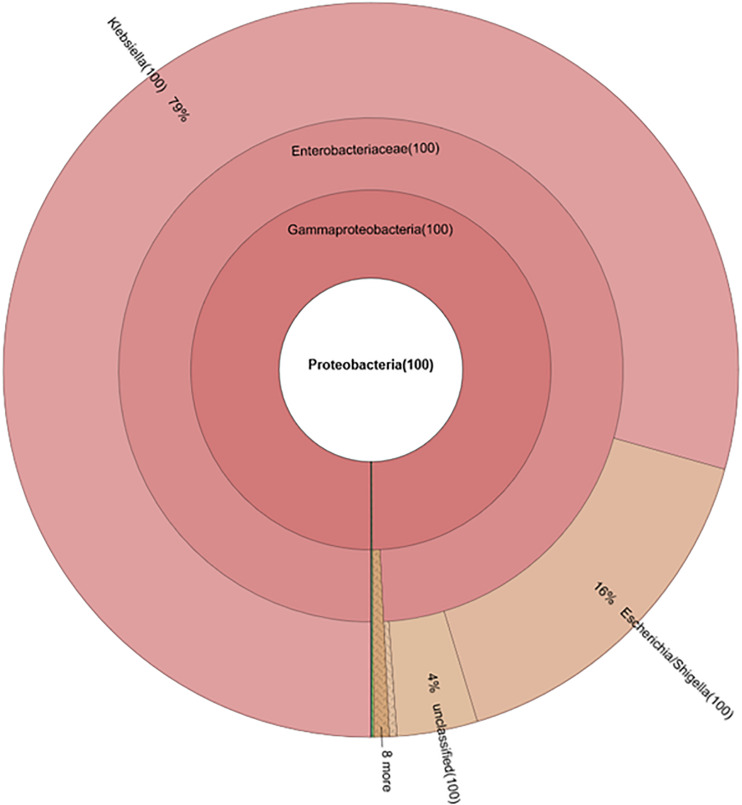
Composition of phylum Proteobacteria in fecal microbiota of NI-Amp-PO pooled sample after antibiotic treatment (D25).

Without seeding the Amp^r^ *E. coli* markers, the difference between antibiotic administration routes could still be recognized in NI-Amp-PO and NI-Amp-IM groups (Figure 5). Oral administration of Amp increased the population of Proteobacteria in NI-Amp-PO chickens, while the microbiota of NI-Amp-IM subjects remained stable with Firmicutes being dominant. Without the inoculation of Amp^r^ marker *E. coli*, the increase of Proteobacteria might be attributed to other resistant strains, such as other members of *Enterobacteriaceae*. As an illustration, *Klebsiella* was detected as the dominant population in the NI-Amp-PO pooled and individual samples (Figure 7 and Supplementary Figure S5) after Ampicillin treatment. It is possible that fecal *Klebsiella* strains in the NI-Amp-PO group also had high tolerance to Amp and thus a growth advantage under Amp selective pressure. Supplementary Figures S4C,D, S6C,D further illustrate the details of diversified but similar trends in additional individual chickens.

### Impact of Drug Administration Routes on Opportunistic Pathogens

Phylogenic analysis at the family level showed that the microbiota shifts after antibiotic administration were mainly induced by the increase of family *Enterobacteriaceae* (Figure 4B). The Amp-PO group had much higher abundance of *Enterobacteriaceae* (ranged from 13.24 to 99.59%, average 54.80 ± 28.57%) than Amp-IM (ranged from 0.05 to 31.03%, average 9.99 ± 14.54%) and control (ranged from 0 to 0.05%, average 0.02 ± 0.02%) groups. The population of *Lactobacillaceae* decreased accordingly, from 80.69 ± 24.16% (ranged from 45.18 to 97.43%) in control, to 50.52 ± 37.50% (ranged from 0.98 to 96.51%) in Amp-IM and 4.00 ± 5.05% (ranged from 0.02 to 12.66%) in Amp-PO (Figure 4C). Supplementary Figure S4A further illustrated in detail by individual subjects treated with oral Amp, that along with the switch of dominant phylum from Firmicutes to Proteobacteria, there was a significant surge of *Escherichia/Shigella* (ranged from 88.88 to 99.90%, average 95.64 ± 5.92%) in Proteobacteria. In the phylum of Firmicutes, the changes also included the reduction of *Lactobacillus* (ranged from 0.28 to 49.96%, average 24.56 ± 24.86%) and the increase of *Clostridium* (ranged from 12.50 to 86.08%, average 34.35 ± 44.98%). Likewise, Supplementary Figure S4B illustrates that while Amp by muscle injection had mild impact on gut microbiota by retained the dominance of Firmicutes at the phylum level, within Firmicutes the reduction of *Lactobacillus* (from 98.27 to 16.42% in chicken 1, from 42.53 to 1.38% in chicken 2) was accompanied by the rise of *Clostridium* (from 0.09% to 9.13% in chicken 1, and from 3.43% to 26.06% in chicken 2) in 2 of the 3 subjects.

Without prior seeding of the *bla*_CMY–2_^+^ *E. coli* marker strains, the dominance of Firmicutes (from 84.82 ± 23.49% on D20, to 57.00 ± 48.89% on D25) was retained in chickens that received oral Amp treatment, but the treatment triggered the increase of Proteobacteria 14.22 ± 24.21% on D20, to 60.67 ± 38.22% on D25, largely due to the rise of *Klebsiella* and *Escherichia/Shigella* in the phylum, as well as the decrease of *Lactobacillus* (from 66.27 ± 29.80% on D20, to 4.64 ± 6.46% on D25) and the rise of *Clostridium* (up to 24.13%), *Enterococcus* (up to 48.09%), etc. in Firmicutes (Supplementary Figures S4C, S5). Likewise, an even milder impact was observed in non-seeded chickens that received Amp by muscle injection (Supplementary Figure S4D).

### Enhanced Impact of Drug Administration Associated With Oral/Environmental Microbial Exposure

As illustrated above, chickens seeded with the *bla*_CMY–2_^+^ *E. coli* marker strains prior to antibiotic treatment exhibited more profound impact on gut microbiota dysbiosis by drug administration routes than control chickens without seeding. In the last round of the validation study, the experiment was housed in the vacated OARDC turkey facility instead of the chicken facility. As illustrated in Supplementary Figure S6E, at D25, even the fecal microbiota of control chickens that received neither seeding of the marker *E. coli* nor Amp treatment substantially differed from the normal fecal microbiota presented in Supplementary Figures S3, S4. Although Firmicutes remained dominant (69.31 ± 21.84%), *Clostridium* (up to 46.25%), *Enterococcus* (up to 88.32%) etc. represented the main subpopulation within the phylum instead of *Lactobacillus* (Supplementary Figure S6E). Oral Amp led to absolute dominance of Proteobacteria in (i) all three representative chickens seeded with the *E. coli* marker strains, with 77, 95, and 99% of the population being *Escherichia/Shigella* (Supplementary Figure S6A); and (ii) all three illustrated chickens without *E. coli* seeding, but with 96 and 73% *Escherichia/Shigella* and 99% *Enterobacteriaceae* in the respective subjects (Supplementary Figure S6C). Amp by muscle injection led to the dominance of Proteobacteria in all three representative chickens seeded with the *E. coli* marker strains, but only with 23, 26, and 60% of *Escherichia/Shigella* in each subject, respectively (Supplementary Figure S6B); and 19, 3, and 68% of *Escherichia/Shigella* in chickens without seeding of the *E. coli* marker strains (Supplementary Figure S6D).

## Discussion, Conclusion, and Perspectives

Despite selective pressure facilitating the expansion of AR, the application of antibiotics is still essential for disease treatment and prevention in both human and food-producing animals. The issue of how to properly address the need to use antibiotics while staving off resistance has been a conundrum for decades. Increasing evidence in the past decade on the correlation between disrupted gut microbiota and non-communicable diseases (Blaser, 2016) as well as host immune functions (Gopalakrishnan et al., 2018; Dhakal et al., 2019) has raised further concerns on the application of antibiotics beyond AR.

It is encouraging that effective reduction of fecal AR and gut microbial disruption as a result of shifting drug administration from oral to injection, exemplified by Amp, tetracycline and vancomycin, has now been illustrated in mice by multiple teams (Zhang et al., 2013; Isaac et al., 2017). Combined with using drugs with reduced impacts on host gut microbiota, this advancement represents a novel plausible direction for mitigating AR while combating gut microbiota disruption and protecting host health. This advancement is further supported by a range of clinical evidence. For instance, vancomycin was marketed in the late 1960s, initially by injection. Oral vancomycin was introduced in the U.S. around 1985, and vancomycin resistant enterococci (VRE) began to surge in the early 1990s. However, despite heavy vancomycin usage at the University of California San Francisco medical center, Luber et al. (1996) reported that the incidences of VRE were extremely low in their facility, as the drug had been primarily delivered by intravenous injection and the use of oral vancomycin was strictly limited. Furthermore, although vancomycin has been used in China since the 1970s, and the total utility of vancomycin-related products in China by 2006 was already around 20% of the vancomycin produced worldwide (China Data Center for Food and Drug Administration, 2008), the prevalence of VRE in China by 2017 was still less than 2% in clinical isolates (Huang et al., 2019). In comparison, the prevalence of VRE in the U.S. was around 30% in 2013 (Faron et al., 2016). While oral vancomycin is still unavailable in China, it has been a recommended treatment option for *Clostridium difficile* infections in the U.S. Morjaria et al. (2019) further illustrated that although a number of antibiotics, including oral vancomycin treatment, induced loss of obligate anaerobic bacteria in gut microbiota of patients, vancomycin by intravenous injection had little impact.

Despite the aforementioned evidence, further demonstration of the broad impact of antibiotic administration route on hosts in conventional settings is essential to translate laboratory findings and clinical observations into practical solutions. This study assessed the impact of drug administration routes using poultry raised in a caged production system. Benefits include controllable risk factors, sufficient numbers of subjects for repeatability, and a range of diversity among individuals. ESBL *E. coli* isolates were examined in the study because β-lactam antibiotics (e.g., penicillin, Amp, and cephalosporin) have been used in the poultry industry to treat or prevent infections by Gram-positive bacteria (Commission of Chinese Veterinary Pharmacopoeia, 2010; Roth et al., 2019). ESBL/AmpC *Enterobacteriaceae* is now prevalent in poultry production systems even without antibiotic applications (Baron et al., 2018; Daehre et al., 2018a; Apostolakos et al., 2019). Among the different types of ESBL-/AmpC-producing *Enterobacteriaceae* found in poultry, *E. coli* harboring the *bla*_CMY–2_ gene was frequently detected (Daehre et al., 2018b; Apostolakos et al., 2019; Seo et al., 2019). Despite of its prevalence, the relatively low abundance of *bla*_CMY–2_ in poultry made it a practical target for the intended assessment.

Data from this study clearly illustrated the critical impact of drug administration routes on gut resistome and microbiota in hosts. Compared to the up to 5-log reduction of the fecal AR gene pool observed in the mouse model (Zhang et al., 2013), the impact of changing Amp administration route from oral to injection on the *bla*_CMY–2_ gene pool itself, assessed by real-time PCR, was insignificant. However, the changes in resistome were still evident. The increase of multidrug-resistance genes and Amp^r^ genes by Amp-PO was especially more palpable than those by Amp-IM. This finding could be primarily due to resistant bacteria with multidrug resistance and Amp^r^ genes beyond *bla*_CMY–2_ already being abundant in the ecosystem and hosts, as detected in the natural gut microbiota of young chicks without experimental manipulation. Thus, there was a limited niche and advantage in the gut for the seeded *bla*_CMY–2_^+^ *E. coli* marker strains to rise. The structural difference between birds (urine and feces both excreted through the cloaca) and mammals (excretion through urinary and GI tracts) might have also contributed to the reduced difference in fecal AR gene pools between the two drug administration routes. Regardless, resistome data still clearly illustrated that oral Amp administration had larger impact than injection on related multidrug-resistance and Amp resistance-determining genes. The changes in other AR genes likely were due to indirect co-selection of multidrug-resistant bacteria, as well as the reduction of Amp-susceptible bacteria that happened to carry other AR genes, instead of the direct selective impact by Amp.

The impact of drug administration routes was most prominent on gut microbiota disruption. Amp administration, especially by oral delivery, flipped the profile from Firmicutes-occupied to dominance by Proteobacteria, and significantly reduced *Lactobacillus*, along with an increase of *Enterococcus, Clostridium* in Firmicutes, and the surge of *Escherichia/Shigella*, and *Klebsiella* etc. in Proteobacteria. It is particularly worth mentioning that many of the increased subpopulations belong to opportunistic pathogens. This finding is critically important because the impact of drug administration route on the surge of opportunistic pathogens in gut microbiota is likely also applicable to people receiving drug treatment. The accumulation of AR bacteria and opportunistic pathogens in human and animal guts, and their subsequent dissemination through feces, have significant public health implications.

Results from this study further illustrated the critical contribution of the original host gut microbiota profile to the outcome of gut microbiota disruption during antibiotic treatment. Gut microbiota disruption was much more obvious in chickens seeded with the Amp^r^ *E. coli* marker strains than in control chickens without prior inoculation. This finding was true for Amp by both oral and injection administration (Supplementary Figures S4, S6). The microbiota disruption was more extreme in chickens raised in the turkey facility than those raised in the poultry facility because the chickens in the turkey facility already had an AR-rich gut microbiota, likely due to increased exposure in the less sanitized environment. This finding is also consistent with a previous report using lab mice from controlled animal facility with enhanced sanitation condition (Zhang et al., 2013). Without prior seeding of the AR bacteria, targeted AR genes were not observed even after 5 days of Amp by oral or injection. Furthermore, the integrity of the gut microbiota of the experimental mice that received Amp by injection was retained, similar to the control mice with no antibiotic treatment.

This consistent finding in multiple host models (poultry and mice) has further implications for human medicine and food animal production beyond drug administration. With the intention to treat various diseases or to establish healthy host immune functions, microbiota transplantation has become a popular medical practice in recent years. Similar practices are currently being examined in food animal production. But data from this study support a recent warning by the FDA (DeFilipp et al., 2019; U.S. Food and Drug Administration, 2019). Without proper screening of AR and other risk factors in donor microbiota, introducing and establishing microbiota in recipients (human or animals) through transplantation or environmental exposure may expose them to potential, unintended long-term health risks (Liu and Wang, 2020). Therefore, developing strategies to minimize unnecessary loss of healthy gut microbiota, when possible, becomes especially important.

While muscle injection used to be the mainstream practice in human medicine, individual injection by veterinarian(s) may be impractical in intensive food animal production operations. However, demonstrating the impact of drug administration routes in food animals lays a solid scientific foundation, and motivates further innovations in drug delivery targeted for industrial applications. It is further worth mentioning that over 30 antibiotics used in humans have injectable counterparts, covering almost all types of antibiotics. Besides searching for new antibiotics with preferred pharmacological properties for human and animal applications, it may be productive to re-evaluate existing antibiotics, considering both their oral and injectable forms. A more thorough understanding of their intended and unintended impacts on gut microbiota can direct exploration of derivatives of existing antibiotics, filtered for aimed features. First, however, targeted strategies for intended mitigation, like changing drug administration routes and using drugs with reduced gut microbiome impact, need to be clearly spelled out and disseminated to protect health, lives, and the industry.

## Data Availability Statement

The datasets generated in this study including the metagenomic sequencing and 16S amplicon sequencing data protocol are available under the accession number PRJNA601052 at the Sequence Read Archive.

## Ethics Statement

The animal study was reviewed and approved by The Ohio State University IACUC committee.

## Author Contributions

HW designed and supervised the study, received the grants, and responsible for the overall manuscript preparation. YZ conducted most of the experiment, data analysis and wrote the draft of the manuscript. LZ, YL, YH, HY, L-JW, and HA participated in the animal experiment. LZ, ZW, and HY participated in the data analysis. JZ was co-PI of the GII grant and assisted with the study design. All authors have read and approved the submitted version.

## Conflict of Interest

The authors declare that the research was conducted in the absence of any commercial or financial relationships that could be construed as a potential conflict of interest.

## Footnotes

1 https://github.com/FelixKrueger/TrimGalore

## Supplementary Material

FIGURE S1 Experimental flow chart.

FIGURE S2 Profiles of pooled microbiota and composition of *Enterobacteriaceae* at D20 before Amp treatment. Inoculation of Amp^r^ *E. coli* had little impact on the average microbiota profile of chicken feces. *Escherichia/Shigella* was the major population in *Enterobacteriaceae*, with low abundance of *Klebsielar, Providencia, Enterobacter* and other species. **(A)** Inoculated; **(B)** non-inoculated.

FIGURE S3 Representative microbiota profiles of individual chickens at D20 before Amp treatment. **(A)** Amp-PO; **(B)** Amp-IM; **(C)** NI-Amp-PO; **(D)** NI-Amp-IM. Each chat represents fecal microbiota profile of one chicken.

FIGURE S4 Representative microbiota profiles of individual chickens at D25 after Amp treatment. **(A)** Amp-PO; **(B)** Amp-IM; **(C)** NI-Amp-PO; **(D)** NI-Amp-IM. Each chat represents fecal microbiota profile of one chicken.

FIGURE S5 The dominance of other *Enteribacteriaceae* in NI-Amp-OP group at D25 after Amp tratment. Each chat represents fecal microbiota profile of one chicken.

FIGURE S6 Representative profiles of fecal microbiota at D25 after Amp treatment^∗^. **(A)** Amp-PO; **(B)** Amp-IM; **(C)** NI-Amp-PO; **(D)** NI-Amp-IM; **(E)** Control. Each chat represents fecal microbiota profile of one chicken. ^∗^Chickens were raised at the turkey teaching farm.

TABLE S1 Composition of chicken feed (P10109 standard diet).

TABLE S2 Abundance of antibiotic-resistance gene types in fecal microbiota of experimental chickens.

TABLE S3 Abundance of antibiotic-resistance genes in fecal microbiota of experimental chickens.
