# Supplementary material for: Antibiotic Administration Routes and Oral Exposure to Antibiotic Resistant Bacteria as Key Drivers for Gut Microbiota Disruption and Resistome in Poultry
Source: Front Microbiol. 2020 Jul 7;11:1319. doi: 10.3389/fmicb.2020.01319 (PMC7358366; doi:10.3389/fmicb.2020.01319)

Supplemental Figure S2. Profiles of pooled microbiota and composition of *Enterobacteriaceae* at D20 before Amp treatment. Inoculation of Amp^{r} *E.coli* had little impact on the average microbiota profile of chicken feces. *Escherichia/Shigella* was the major population in *Enterobacteriaceae*, with low abundance of *Klebsielar*, *Providencia*, *Enterobacter* and other species. A. Inoculated; B. Non-inoculated.

A.

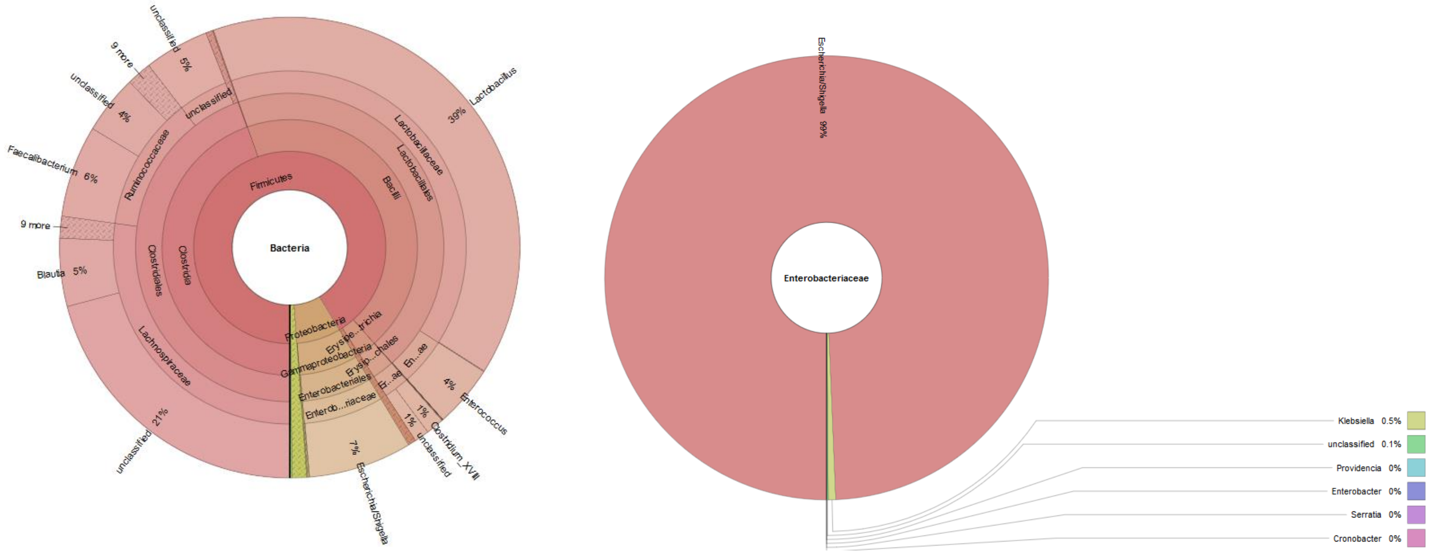

B.

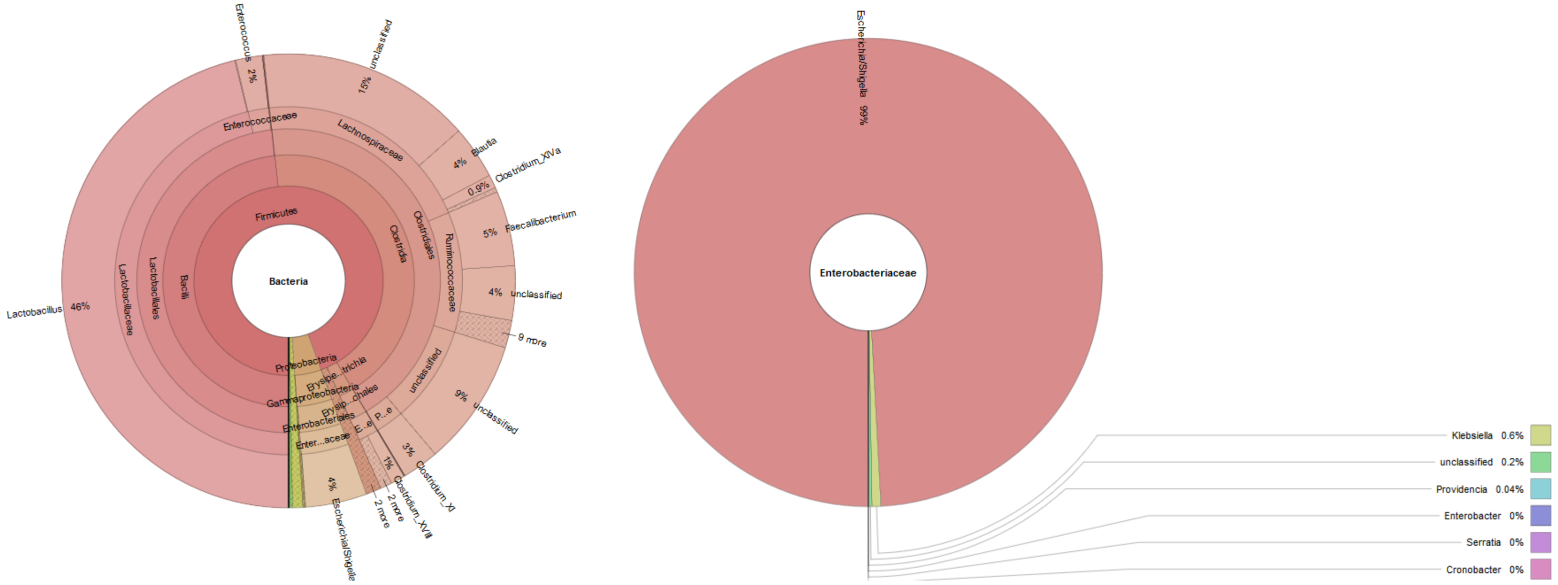

Supplement: FIGURE S1 — Experimental flow chart. [file Data_Sheet_1.zip › Figure S2.pdf]
