# Supplementary material for: Antibiotic Administration Routes and Oral Exposure to Antibiotic Resistant Bacteria as Key Drivers for Gut Microbiota Disruption and Resistome in Poultry
Source: Front Microbiol. 2020 Jul 7;11:1319. doi: 10.3389/fmicb.2020.01319 (PMC7358366; doi:10.3389/fmicb.2020.01319)
Supplement: FIGURE S1 — Experimental flow chart. [file Data_Sheet_1.zip › Figure S3.pdf]

Supplemental Figure S3. Representative microbiota profiles of individual chickens at D20 before Amp treatment. A. Amp-PO; B. Amp-IM; C. NI-Amp-PO; D. NI-Amp-IM. Each chat represents fecal microbiota profile of one chicken.

A.

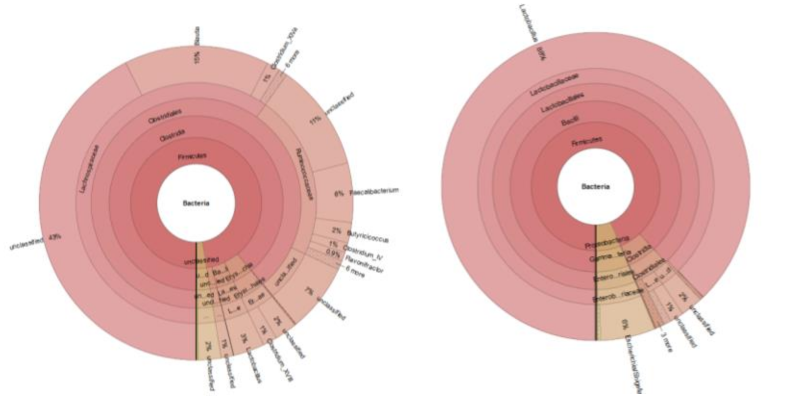

B.

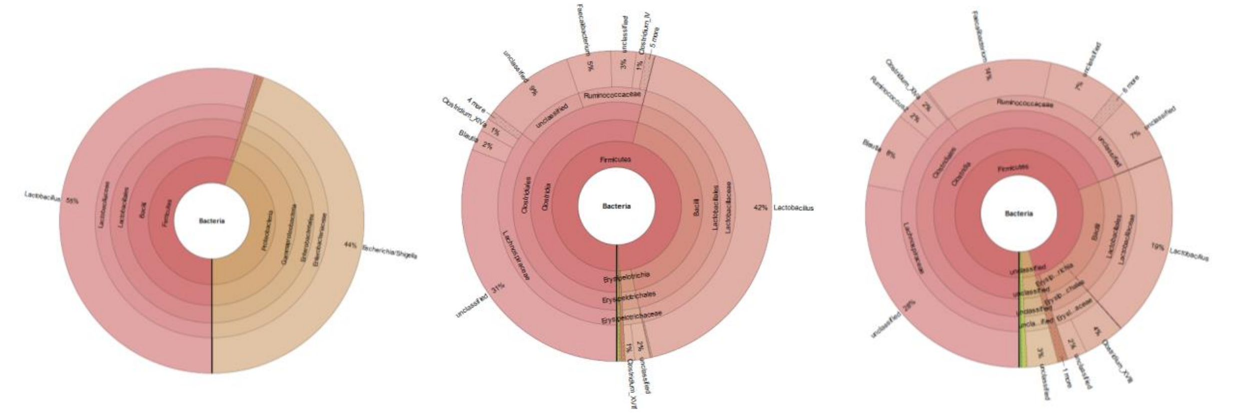

C.

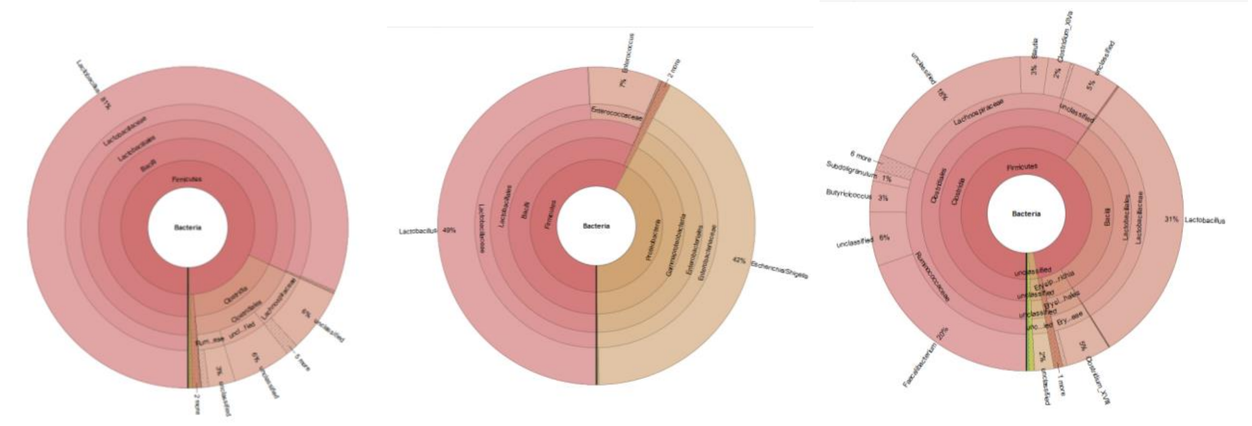

D.

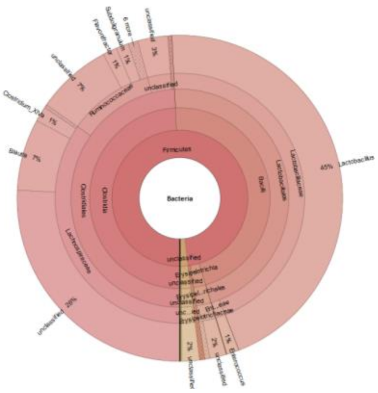
Bacteria
Firmicutes
Clostridia
Clostridiales
Ruminococcaceae
Lachnospiraceae
Bacilli
Lactobacillales
Lactobacillaceae
45% Lactobacillus
Blautia 7%
Clostridium_XlVa 1%
unclassified 7%
unclassified 26%

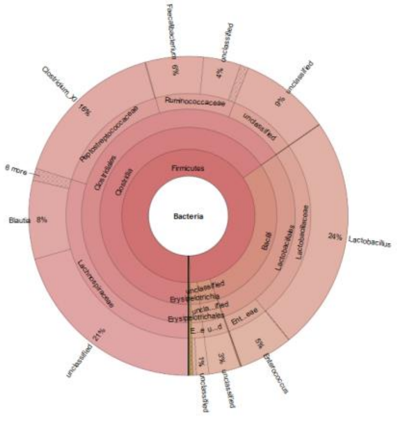
Bacteria
Firmicutes
Clostridia
Clostridiales
Ruminococcaceae
Peptostreptococcaceae
Lachnospiraceae
Bacilli
Lactobacillales
Lactobacillaceae
24% Lactobacillus
Blautia 8%
Clostridium_XI 15%
Faecalibacterium 6%
6% unclassified
unclassified 21%
8 more
5% Enterococcus

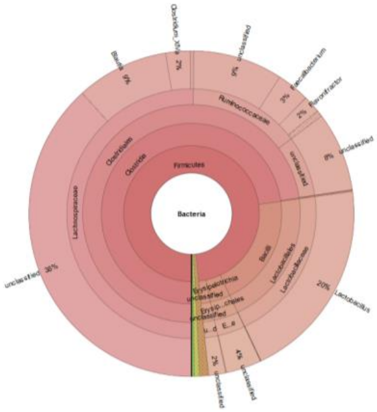
Bacteria
Firmicutes
Clostridia
Clostridiales
Ruminococcaceae
Lachnospiraceae
Bacilli
Lactobacillales
Lactobacillaceae
20% Lactobacillus
unclassified 38%
Blautia 9%
8% unclassified
