# Supplementary material for: Antibiotic Administration Routes and Oral Exposure to Antibiotic Resistant Bacteria as Key Drivers for Gut Microbiota Disruption and Resistome in Poultry
Source: Front Microbiol. 2020 Jul 7;11:1319. doi: 10.3389/fmicb.2020.01319 (PMC7358366; doi:10.3389/fmicb.2020.01319)

Supplemental Figure S4. Representative microbiota profiles of individual chickens at D25 after Amp treatment. A: Amp-PO; B: Amp-IM; C NI-Amp-PO; D: NI-Amp-IM. Each chat represents fecal microbiota profile of one chicken.

A.

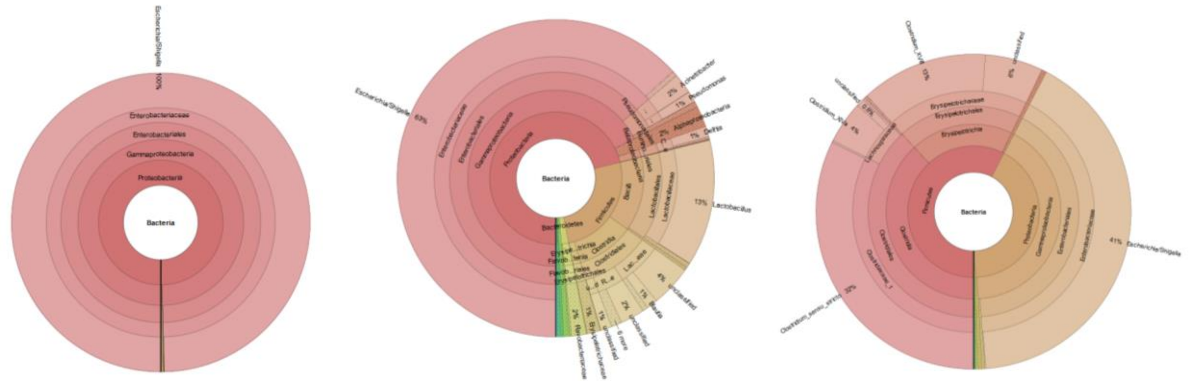

B.

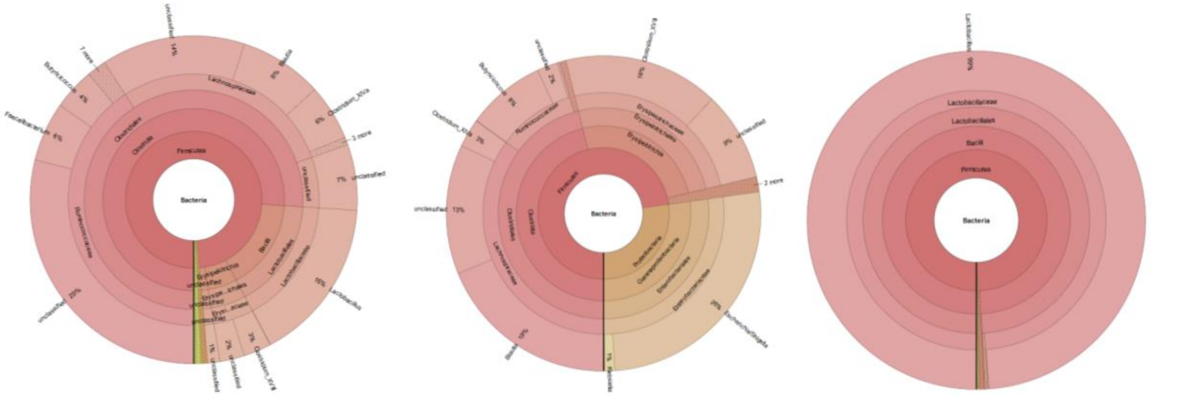

C.

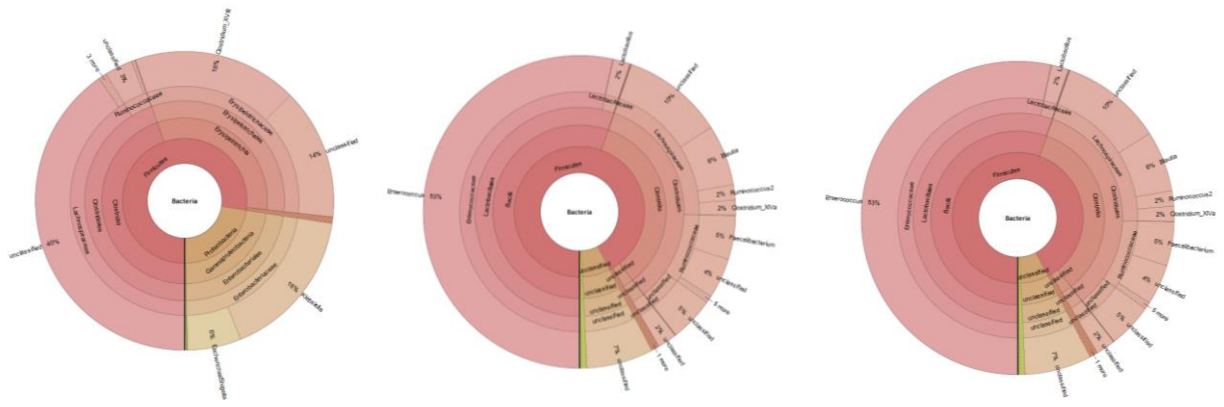

D.

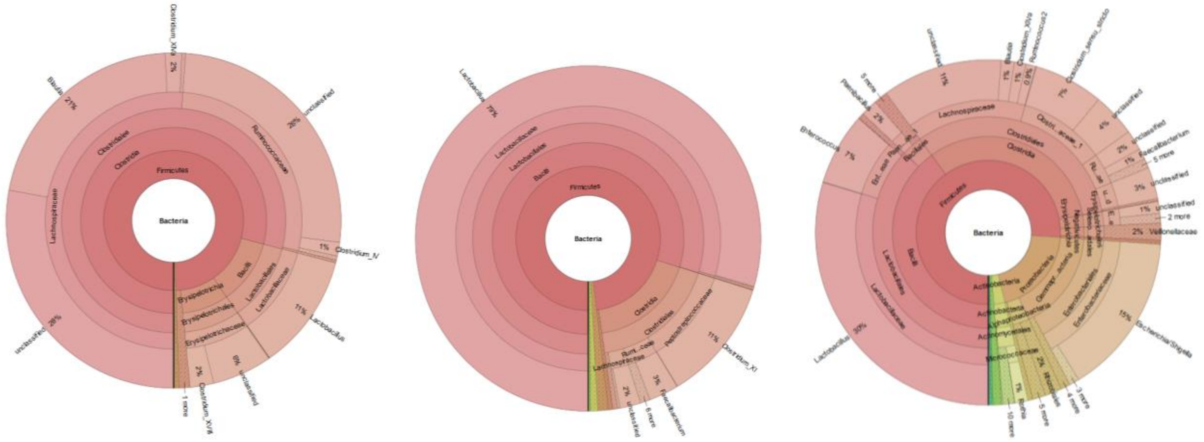

Supplement: FIGURE S1 — Experimental flow chart. [file Data_Sheet_1.zip › Figure S4.pdf]
