# Supplementary material for: Antibiotic Administration Routes and Oral Exposure to Antibiotic Resistant Bacteria as Key Drivers for Gut Microbiota Disruption and Resistome in Poultry
Source: Front Microbiol. 2020 Jul 7;11:1319. doi: 10.3389/fmicb.2020.01319 (PMC7358366; doi:10.3389/fmicb.2020.01319)

Figure S5. The dominance of other *Enteribacteriaceae* in NI-Amp-OP group at D25 after Amp tratment. Each chat represents fecal microbiota profile of one chicken.

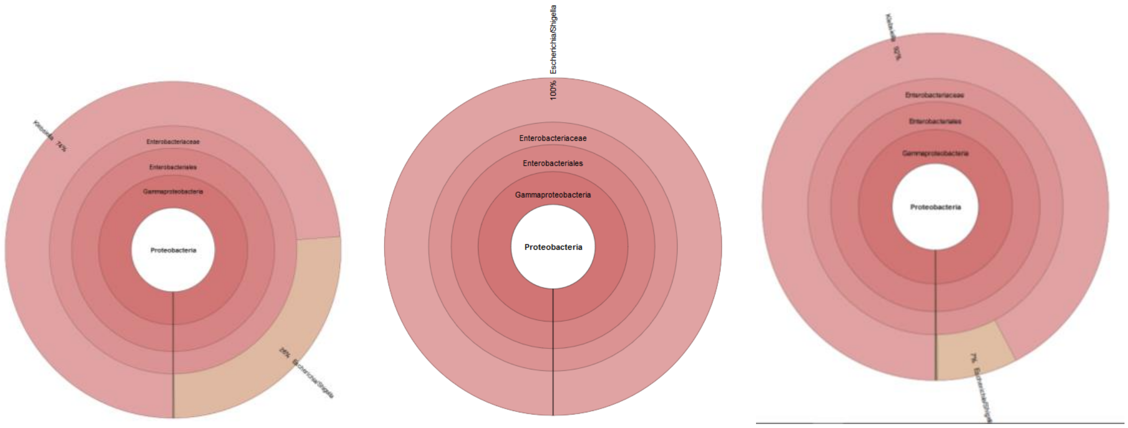

Supplement: FIGURE S1 — Experimental flow chart. [file Data_Sheet_1.zip › Figure S5.pdf]
