# Supplementary material for: Antibiotic Administration Routes and Oral Exposure to Antibiotic Resistant Bacteria as Key Drivers for Gut Microbiota Disruption and Resistome in Poultry
Source: Front Microbiol. 2020 Jul 7;11:1319. doi: 10.3389/fmicb.2020.01319 (PMC7358366; doi:10.3389/fmicb.2020.01319)
Supplement: FIGURE S1 — Experimental flow chart. [file Data_Sheet_1.zip › Figure S6.pdf]

Supplemental Figure S6. Representative profiles of fecal microbiota at D25 after Amp treatment*. A. Amp-PO; B. Amp-IM; C. NI-Amp-PO. D. NI-Amp-IM; E. Control. Each chat represents fecal microbiota profile of one chicken. *Chickens were raised at the turkey teaching farm.

A.

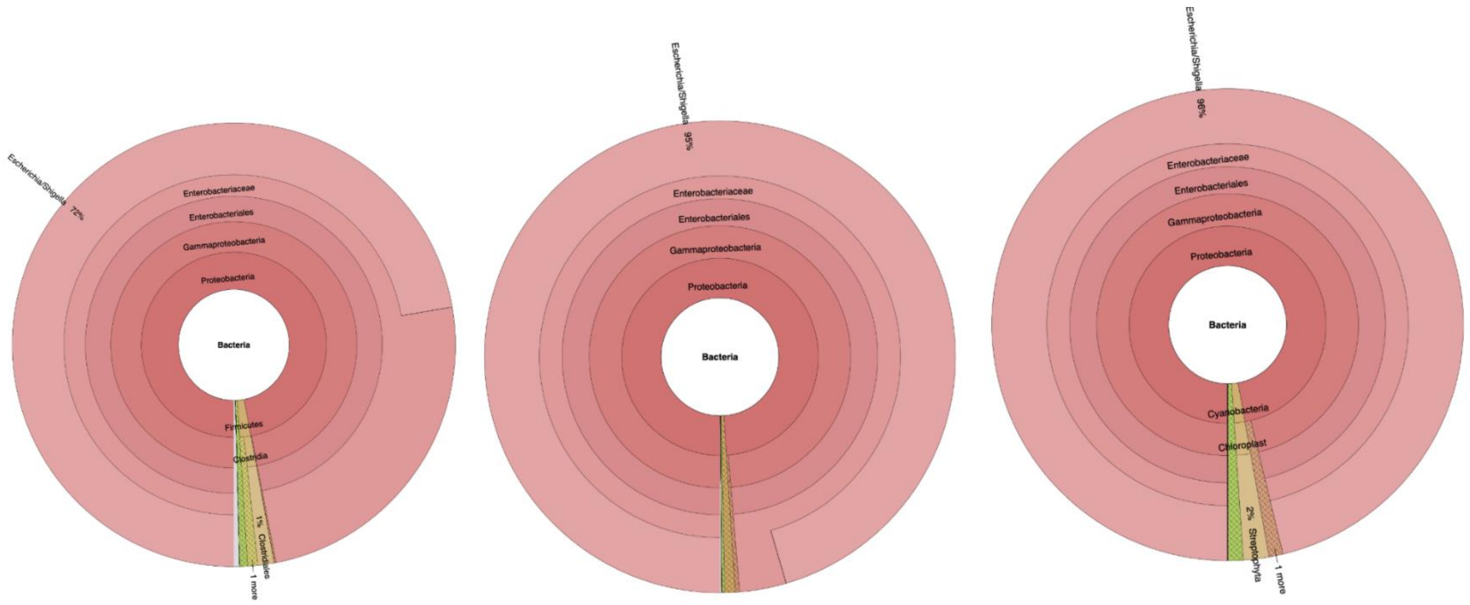

B.

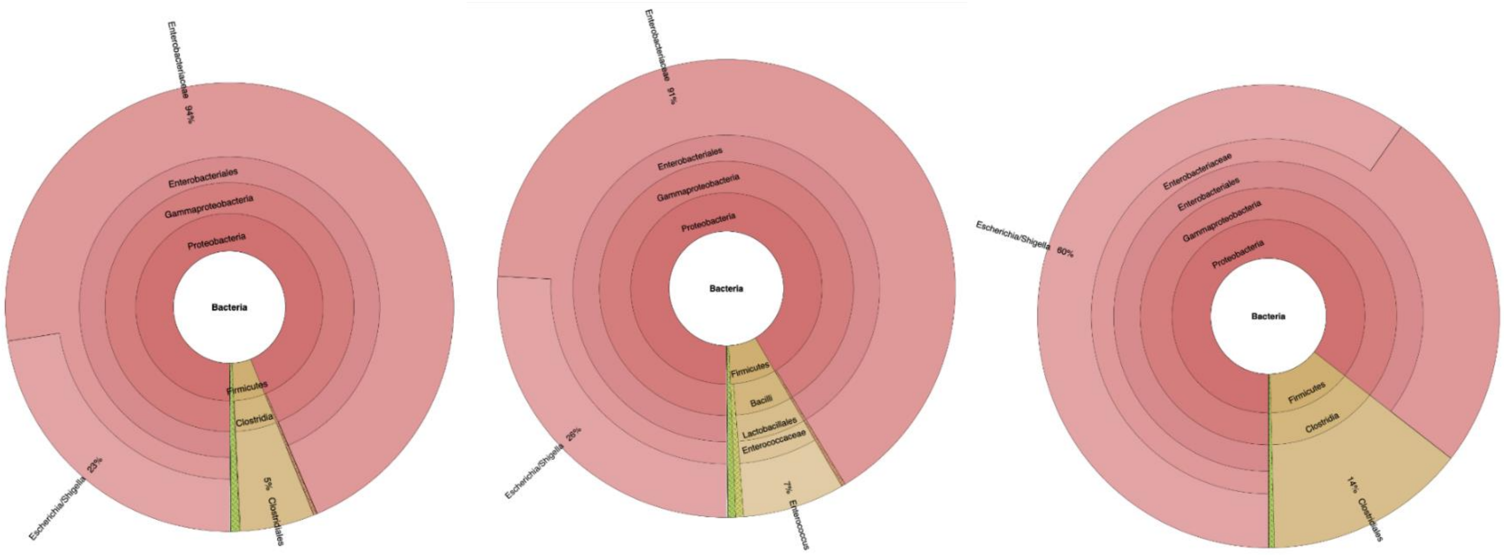

C.

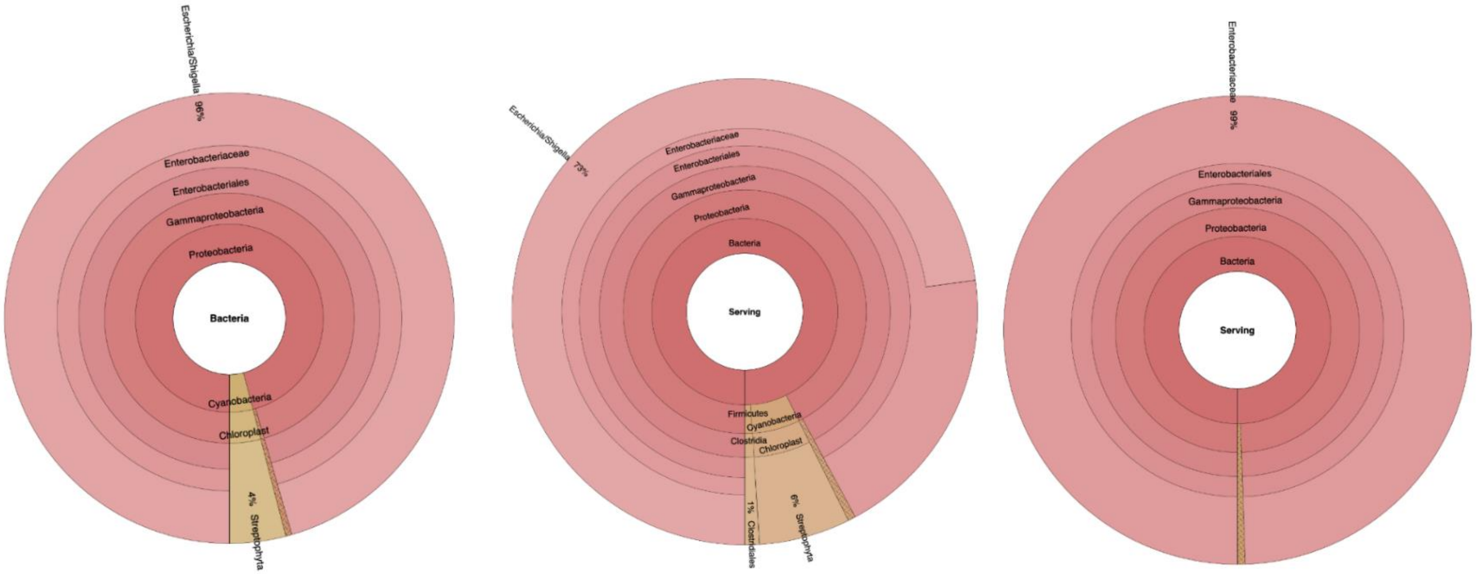
Escherichia/Shigella 96%
Enterobacteriaceae
Enterobacteriales
Gammaproteobacteria
Proteobacteria
Bacteria
Cyanobacteria
Chloroplast
4% Streptophyta
Escherichia/Shigella 73%
Enterobacteriaceae
Enterobacteriales
Gammaproteobacteria
Proteobacteria
Bacteria
Serving
Firmicutes
Cyanobacteria
Clostridia
Chloroplast
1% Clostridiales
6% Streptophyta
Enterobacteriaceae 99%
Enterobacteriales
Gammaproteobacteria
Proteobacteria
Bacteria
Serving

D.

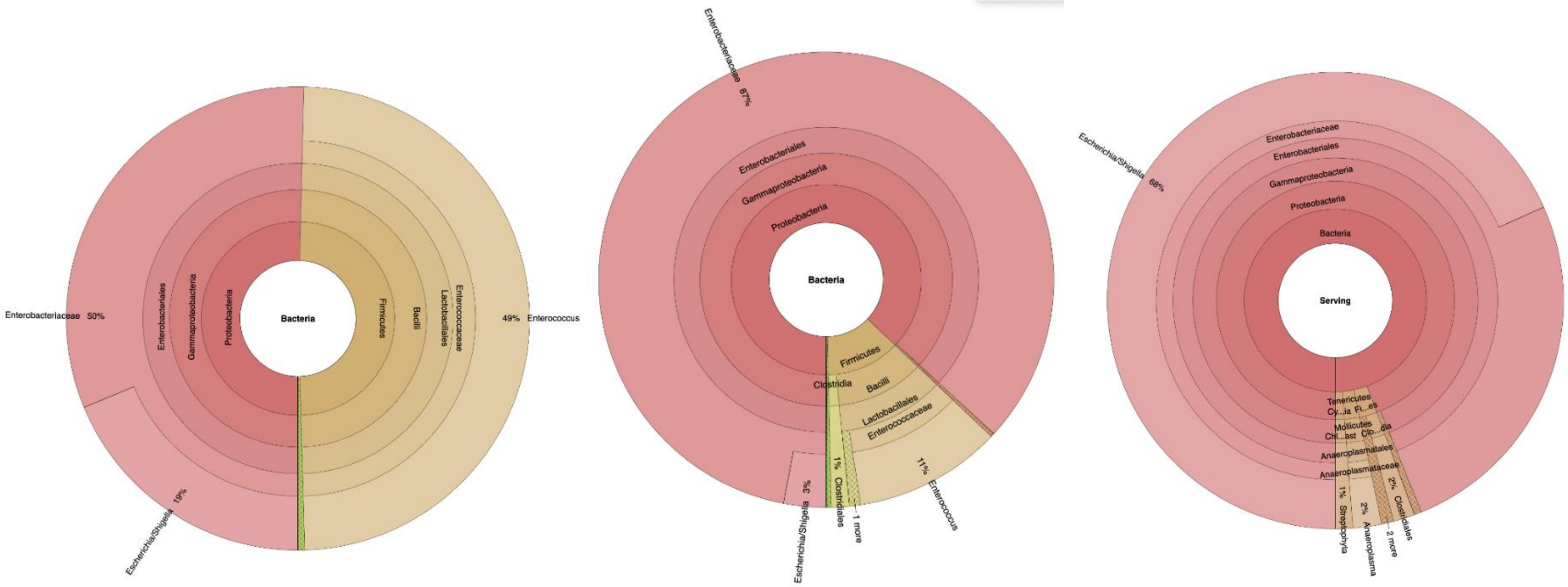
Enterobacteriaceae 50%
Enterobacteriales
Gammaproteobacteria
Proteobacteria
Bacteria
Firmicutes
Bacilli
Lactobacillales
Enterococcaceae
49% Enterococcus
Escherichia/Shigella 19%
Enterobacteriaceae 87%
Enterobacteriales
Gammaproteobacteria
Proteobacteria
Bacteria
Firmicutes
Clostridia
Bacilli
Lactobacillales
Enterococcaceae
11% Enterococcus
Escherichia/Shigella 3%
1% Clostridiales
1 more
Escherichia/Shigella 68%
Enterobacteriaceae
Enterobacteriales
Gammaproteobacteria
Proteobacteria
Bacteria
Serving
Tenericutes
Mollicutes
Anaeroplasmatales
Anaeroplasmataceae
1% Streptophyta
2% Anaeroplasma
2% Clostridiales
2 more

E.

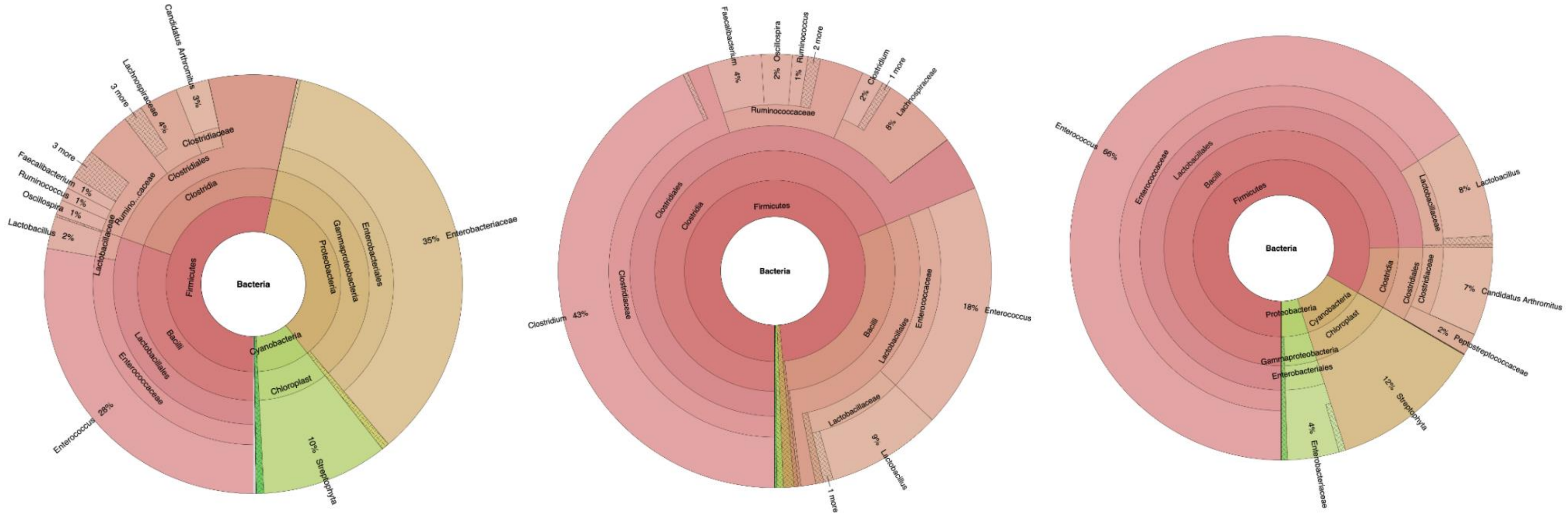
Bacteria
Firmicutes
Bacilli
Lactobacillales
Enterococcaceae
Enterococcus 28%
Clostridia
Clostridiales
Clostridiaceae
Candidatus Arthromitus 3%
Lachnospiraceae 4%
3 more
Lactobacillaceae
Lactobacillus 2%
Faecalibacterium 1%
Ruminococcus 1%
Oscillospira 1%
Proteobacteria
Gammaproteobacteria
Enterobacteriales
35% Enterobacteriaceae
Cyanobacteria
Chloroplast
10% Streptophyta
Bacteria
Firmicutes
Clostridia
Clostridiales
Clostridiaceae
Clostridium 43%
Ruminococcaceae
Faecalibacterium 4%
2% Oscillospira
1% Ruminococcus
2 more
2% Clostridium
1 more
8% Lachnospiraceae
Bacilli
Lactobacillales
Enterococcaceae
18% Enterococcus
Lactobacillaceae
9% Lactobacillus
1 more
Bacteria
Firmicutes
Bacilli
Lactobacillales
Enterococcaceae
Enterococcus 66%
Lactobacillaceae
8% Lactobacillus
Clostridia
Clostridiales
Clostridiaceae
7% Candidatus Arthromitus
2% Peptostreptococcaceae
Proteobacteria
Gammaproteobacteria
Enterobacteriales
4% Enterobacteriaceae
Cyanobacteria
Chloroplast
12% Streptophyta
