# Supplementary figures and images for: Antibiotic Administration Routes and Oral Exposure to Antibiotic Resistant Bacteria as Key Drivers for Gut Microbiota Disruption and Resistome in Poultry
Source: Front Microbiol. 2020 Jul 7;11:1319. doi: 10.3389/fmicb.2020.01319 (PMC7358366; doi:10.3389/fmicb.2020.01319)

Supplemental Figure S1. Experimental flowchart.

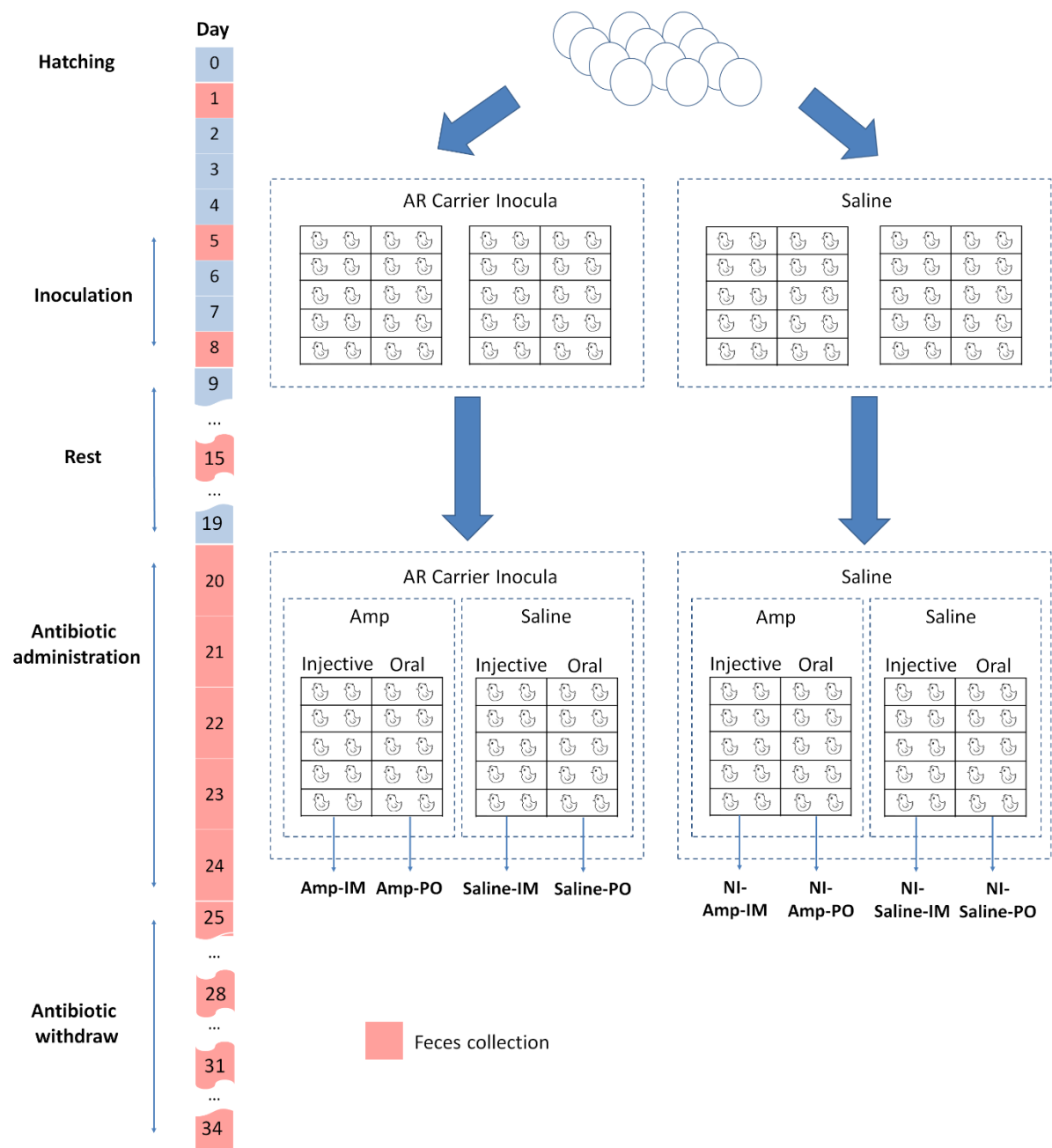

Supplement: FIGURE S1 — Experimental flow chart. [file Data_Sheet_1.zip › Figure S1.pdf]
