# Supplementary material for: Antibiotic Administration Routes and Oral Exposure to Antibiotic Resistant Bacteria as Key Drivers for Gut Microbiota Disruption and Resistome in Poultry
Source: Front Microbiol. 2020 Jul 7;11:1319. doi: 10.3389/fmicb.2020.01319 (PMC7358366; doi:10.3389/fmicb.2020.01319)
Supplement: TABLE S1 — Composition of chicken feed (P10109 standard diet). [file Table_1.DOCX]

Table S1. Composition of chicken feed (P10109 standard diet).

| Component | % (weight/weight) |
| --- | --- |
| Corn, ground, rolled | 41.55 |
| Soybean meal, 47% | 44.4 |
| Meat and bone meal, 50% (pork) | 5 |
| Fat (true energy formula fat) | 2.9 |
| Salt, fine | 0.4 |
| Limestone feeding grade | 0.7 |
| Copper sulfite, fine, 25.2% | 0.05 |
| Amprolium 2.5 | 1 |
| Dicalcium phosphate 18.5% | 2.85 |
| Selenium, 90.8 mg/lb | 0.1 |
| DL Methionine, 99% | 0.25 |
| Choline Chloride 60% | 0.15 |
| Akey starter breeder | 0.5 |
| Lysine 99% | 0.15 |
