# Supplementary material for: Antibiotic Administration Routes and Oral Exposure to Antibiotic Resistant Bacteria as Key Drivers for Gut Microbiota Disruption and Resistome in Poultry
Source: Front Microbiol. 2020 Jul 7;11:1319. doi: 10.3389/fmicb.2020.01319 (PMC7358366; doi:10.3389/fmicb.2020.01319)
Supplement: TABLE S2 — Abundance of antibiotic-resistance gene types in fecal microbiota of experimental chickens. [file Table_2.DOCX]

**Supplemental Table 2**. Relative abundance of antibiotic resistance gene types in fecal microbiota of experimental chickens.

| AR Type | 16S Normalized Read Count | | | |
| --- | --- | --- | --- | --- |
|  | Amp-PO | Amp-IM | Sham | Control-D5 |
| aminoglycoside | 0.502756762 | 0.158372408 | 0.043735039 | 0.041461333 |
| aminoglycoside:aminocoumarin | 0 | 0 | 0.004812194 | 0.00011809 |
| bacitracin | 0.533689816 | 0.104850008 | 0.080378548 | 0.11351843 |
| beta_lactam | 0.51517608 | 0.226628543 | 0.014765017 | 0.056471855 |
| diaminopyrimidine | 0 | 0 | 0.00032183 | 0.006279693 |
| chloramphenicol | 0.014838422 | 0.00020798 | 0 | 0 |
| fluoroquinolone | 0 | 0 | 0.01379481 | 0.070243937 |
| fluoroquinolone,multidrug | 0 | 0 | 0.003488983 | 0 |
| fosfomycin | 0.074031767 | 0.072891713 | 0 | 0.001555439 |
| fosmidomycin | 0.060522156 | 0.043623262 | 0.005578389 | 7.45E-05 |
| fosmidomycin,polymyxin/fosmidomycin | 0.007974283 | 0 | 0 | 0 |
| glycopeptide | 0.488919506 | 0.208781249 | 0.310507731 | 0.489055921 |
| kasugamycin | 0.085439269 | 0.060504699 | 0 | 0 |
| macrolide-lincosamide-streptogramin | 1.293755771 | 0.157529004 | 0.404808664 | 0.94444987 |
| multidrug | 4.252648643 | 2.521887303 | 0.582724336 | 0.645169207 |
| multidrug/fluoroquinolone,multidrug | 0 | 0 | 0 | 0.000104366 |
| nitroimidazole | 0 | 0 | 0.004791324 | 0.00100454 |
| nucleoside | 0 | 0 | 0.001221763 | 0.000199878 |
| peptide | 0.061450254 | 0.04190502 | 0.016097782 | 0.024700088 |
| peptide,polymyxin | 0 | 0 | 0.002604458 | 0 |
| phenicol | 0 | 0 | 0 | 0.003388624 |
| pleuromutilin | 0.019619192 | 0 | 0.00463591 | 0 |
| polymyxin | 0.286295377 | 0.185009119 | 0 | 0 |
| quinolone | 0.430309963 | 0.328880787 | 0 | 0 |
| rifamycin | 0 | 0 | 0.000791702 | 0.003687743 |
| sulfonamide | 0.080645063 | 0.047036335 | 0 | 0 |
| tetracycline | 1.55254533 | 0.671399509 | 0.178009273 | 0.31075979 |
| trimethoprim | 0.077583699 | 0.056252511 | 0 | 0 |
| unclassified | 0 | 0 | 0.108432671 | 0.079598519 |
