# Supplementary material for: Antibiotic Administration Routes and Oral Exposure to Antibiotic Resistant Bacteria as Key Drivers for Gut Microbiota Disruption and Resistome in Poultry
Source: Front Microbiol. 2020 Jul 7;11:1319. doi: 10.3389/fmicb.2020.01319 (PMC7358366; doi:10.3389/fmicb.2020.01319)
Supplement: TABLE S3 — Abundance of antibiotic-resistance genes in fecal microbiota of experimental chickens. [file Table_3.DOCX]

**Supplemental Table 3**. Relative abundance of antibiotic resistance genes in fecal microbiota of experimental chickens.

| ARG group | 16S Normalized Read Count | | | |
| --- | --- | --- | --- | --- |
|  | Amp-PO | Amp-IM | Sham | Control-D5 |
| AAC(6')-I | 0 | 0 | 0.009421592 | 0.015801803 |
| AAC(6')-IAD | 0.000240562 | 0 | 0 | 0 |
| AAC(6')-IE-APH(2'')-IA | 0.077429922 | 0 | 0 | 0 |
| AAC(6')-IP | 0.002140734 | 0 | 0 | 0 |
| AAD(6) | 0.006275538 | 0.007419633 | 0 | 0 |
| AAD(9) | 0 | 0 | 0.000270667 | 0.00012108 |
| AADE | 0 | 0 | 0.016684018 | 0.038008381 |
| ACRA | 0.090907845 | 0.064747139 | 0.002535387 | 0 |
| ACRB | 0.144320867 | 0.110651713 | 0.004613619 | 0.000125163 |
| ACRD | 0.032057652 | 0.022346378 | 0.00102218 | 0 |
| ACRE | 0.063553435 | 0.038010847 | 0 | 0 |
| ACRF | 0.028219772 | 0.009365252 | 0.003134991 | 0 |
| ACRS | 0.04991449 | 0.019428811 | 0.004478315 | 0 |
| ADEC | 0 | 0 | 0 | 0.015006806 |
| ADEG | 0.001919047 | 0.001628163 | 0 | 0 |
| ADP-RIBOSYLATING_TRANSFERASE_ARR | 0 | 0 | 0.000791702 | 0.003687743 |
| AMPC | 0.008382671 | 0.00128871 | 0 | 0 |
| ANT(6)-IA | 0.004304975 | 0.003281545 | 0 | 0 |
| ANT(6)-IB | 0.150516031 | 0.008964714 | 0 | 0 |
| APH(2'')-IE | 0.001956467 | 0 | 0.001609852 | 0 |
| APH(2'')-IG | 0.001282999 | 0.000284104 | 0 | 0 |
| APH(2'')-II | 0 | 0 | 0.006869656 | 0 |
| APH(2'')-IIA | 0.000810993 | 8.08E-05 | 0 | 0 |
| APH(2'')-IV | 0 | 0 | 0.000730622 | 0.001001048 |
| APH(2'')-IVA | 0.014999579 | 0.000802286 | 0 | 0 |
| APH(3''')-III | 0 | 0 | 0.003848552 | 0.000425876 |
| APH(3')-IIIA | 0.008166313 | 0.012707964 | 0 | 0 |
| APHA | 0.000713328 | 0.001081568 | 0 | 0 |
| APHA-3 | 0.001238758 | 0.001313843 | 0 | 0 |
| ARNA | 0.042986296 | 0.023029179 | 0.002604458 | 0 |
| ARNC | 0.048590596 | 0.02990852 | 0 | 0 |
| ARND | 0.02222992 | 0.019450466 | 0 | 0 |
| BACA | 0.034478337 | 0.030117982 | 0.043564561 | 0.0878034 |
| BACTERIAL_REGULATORY_PROTEIN_LUXR | 0 | 0 | 0.001918886 | 0 |
| BACA2 | 0.00077524 | 0 | 0 | 0 |
| BAER | 0.073419613 | 0.046506574 | 0.002895577 | 0.000224862 |
| BAES | 0.059094875 | 0.03761422 | 0.002524104 | 0 |
| BASR | 0.063508447 | 0.044729679 | 0 | 0 |
| BCR | 0.069573532 | 0.046797457 | 0 | 0 |
| BCRA | 0.120941516 | 0.017898526 | 0.032524979 | 0.022719104 |
| BCRD | 0.10287699 | 0.010044503 | 0 | 0 |
| BICYCLOMYCIN-MULTIDRUG_EFFLUX_PROTEIN_BCR | 0 | 0 | 0.002421311 | 0 |
| BIFUNCTIONAL_AMINOGLYCOSIDE_N-ACETYLTRANSFERASE_AND_AMINOGLYCOSIDE_PHOSPHOTRANSFERASE | 0 | 0 | 0.001567638 | 0 |
| BL1_CMY2 | 0.012562429 | 0 | 0 | 0 |
| BL1_EC | 0.007603346 | 0.001675901 | 0 | 0 |
| BL2_LEN | 0 | 0.000118211 | 0 | 0 |
| BLA(AMPC-EC80-8652) | 0.008300356 | 0.001652624 | 0 | 0 |
| BLACMY | 0.097709965 | 0.010914499 | 0 | 0 |
| BLACMY-127 | 0.000151535 | 0 | 0 | 0 |
| BLACMY-2 | 0.003000399 | 0.000253531 | 0 | 0 |
| BLACMY-96 | 0.008879969 | 0.001166242 | 0 | 0 |
| BLALEN | 0.001937956 | 0.001367869 | 0 | 0 |
| BLAOXA | 0.002557387 | 0 | 0 | 0 |
| BLASHV | 0.000763437 | 0.000878138 | 0 | 0 |
| BLASHV-12 | 0 | 9.11E-05 | 0 | 0 |
| BN452_01100 | 0 | 0.00020798 | 0 | 0 |
| BN537_02049 | 0.059457999 | 0.008949579 | 0 | 0 |
| BU55_14045 | 0.001286402 | 0 | 0 | 0 |
| CAMP-REGULATORY_PROTEIN | 0 | 0 | 0.006988781 | 0.000193133 |
| CAT | 0.011708864 | 0 | 0 | 0 |
| CAT_CHLORAMPHENICOL_ACETYLTRANSFERASE | 0 | 0 | 0 | 0.003388624 |
| CATD | 0.002694298 | 0 | 0 | 0 |
| CATS | 0.000583837 | 0 | 0 | 0 |
| CDEA | 0.001256815 | 0 | 0 | 0.000101978 |
| CEOB | 0.002579299 | 0.002594688 | 0 | 0 |
| CLASS_C | 0 | 0 | 0.005153958 | 0 |
| CLOSTRIDIUM_PERFRINGENS_MPRF | 0 | 0 | 0 | 0.004117866 |
| CMY-115 | 0.000291407 | 0 | 0 | 0 |
| CMY-2 | 0.000575834 | 8.87E-05 | 0 | 0 |
| CMY-20 | 0.023853651 | 0.00242759 | 0 | 0 |
| CMY-25 | 0.004697594 | 0.000583121 | 0 | 0 |
| CMY-4 | 0.001178972 | 0 | 0 | 0 |
| CMY-48 | 0.001394125 | 0 | 0 | 0 |
| CMY-50 | 0.000369504 | 0 | 0 | 0 |
| CMY-51 | 0.004550297 | 0.000347907 | 0 | 0 |
| CMY-71 | 0.007455537 | 0.000925387 | 0 | 0 |
| CMY-73 | 0.005788649 | 0.000671857 | 0 | 0 |
| CMX | 0 | 0 | 0.006268508 | 0 |
| COB(I)ALAMIN_ADENOLSYLTRANSFERASE | 0 | 0 | 0.001929886 | 0 |
| CPXA | 0.095660624 | 0.073196772 | 0.004812194 | 0.00011809 |
| CRP | 0.101888443 | 0.060946985 | 0 | 0 |
| DFRE | 0.077583699 | 0.056252511 | 0.00032183 | 0.006279693 |
| DNA-BINDING_PROTEIN_H-NS | 0 | 0 | 0.002375748 | 0 |
| DNA-BINDING_TRANSCRIPTIONAL_REGULATOR_GADX | 0 | 0 | 0.004285979 | 0 |
| ECW26_04230 | 0.005297331 | 0.000970931 | 0 | 0 |
| EFMA | 0 | 0 | 0 | 0.012861287 |
| EFRA | 0.021689295 | 0.002798274 | 0.005640764 | 0.057203131 |
| EFRB | 0.044305993 | 0.007791659 | 0.019197197 | 0.06872589 |
| EMRA | 0.063123547 | 0.045907498 | 0.002684118 | 0 |
| EMRB | 0.098806011 | 0.065466937 | 0.003857142 | 7.03E-05 |
| EMRB-QACA_FAMILY_MAJOR_FACILITATOR_TRANSPORTER | 0 | 0 | 0.062134626 | 0.058828857 |
| EMRD | 0.07552435 | 0.06110751 | 0.003974139 | 0 |
| EMRE | 0.041254247 | 0.002151439 | 0.003096435 | 0 |
| EMRK | 0.025363902 | 0.00195392 | 0.002045742 | 0 |
| EMRR | 0 | 0 | 0.003423712 | 7.67E-05 |
| EMRY | 0.022845901 | 0.002084721 | 0.001151129 | 0 |
| EPTA | 0.020708588 | 0.015954988 | 0.003988262 | 0 |
| ERMA | 0 | 0 | 0.000245204 | 7.83E-05 |
| ERMB | 0.005028528 | 0.052445752 | 0.054018859 | 0.063402009 |
| ERMG | 0.000615208 | 0.002513851 | 0.004506501 | 0.0024698 |
| ERMT | 0 | 0 | 0.000126182 | 7.37E-05 |
| ESCHERICHIA_COLI_LAMB | 0 | 0 | 0.004526548 | 0 |
| ESCHERICHIA_COLI_MIPA | 0 | 0 | 0.002624819 | 0 |
| EVGA | 0.003848997 | 0 | 0 | 0 |
| EVGS | 0.025650332 | 0.003385271 | 0.001807841 | 0 |
| FOSA2 | 0.000573255 | 0.000616565 | 0 | 0 |
| FOSA3 | 0 | 0.000384981 | 0 | 0 |
| FOSA5 | 0.07451547 | 0.072932866 | 0 | 0 |
| FOSX | 0 | 0 | 0 | 0.001555439 |
| FSR | 0.060522156 | 0.038614296 | 0 | 0 |
| GADE | 0.034970885 | 0.002373483 | 0 | 0 |
| GADW | 0.03354353 | 0.002554584 | 0.00438017 | 0 |
| GADX | 0.035989524 | 0.003225716 | 0 | 0 |
| HMPREF1589_03333 | 0.001755857 | 0 | 0 | 0 |
| KANR | 0 | 7.64E-05 | 0 | 0 |
| KASUGAMYCIN_RESISTANCE_PROTEIN_KSGA | 0 | 0 | 0.00362501 | 0 |
| KDPE | 0.076261456 | 0.046193981 | 0.003616417 | 0.000599633 |
| KLEBSIELLA_PNEUMONIAE_OMPK36 | 0 | 0 | 0.0001558 | 0 |
| KLEBSIELLA_PNEUMONIAE_OMPK37 | 0 | 0 | 0.001399473 | 0 |
| KSGA | 0.085439269 | 0.060504699 | 0 | 0 |
| LEN-10 | 0.000658574 | 0.000808021 | 0 | 0 |
| LEN-11 | 0.003292868 | 0.004793033 | 0 | 0 |
| LEN-14 | 0.000151934 | 0 | 0 | 0 |
| LEN-18 | 0.014332803 | 0.015012789 | 0 | 0 |
| LEN-19 | 0.00157459 | 0.002161571 | 0 | 0 |
| LEN-1-LIKE | 0.000683968 | 0.000302912 | 0 | 0 |
| LEN-2 | 0.000484489 | 0.000776815 | 0 | 0 |
| LEN-21 | 0.000242245 | 0.00055728 | 0 | 0 |
| LEN-22 | 0.005410128 | 0.005674125 | 0 | 0 |
| LEN-4 | 0 | 0.000176269 | 0 | 0 |
| LEN-6 | 0 | 0.001045936 | 0 | 0 |
| LEUO | 0.080645063 | 0.047036335 | 0 | 0 |
| LLMA | 0.054073711 | 0.006613575 | 0 | 0 |
| LLMA_23S_RIBOSOMAL_RNA_METHYLTRANSFERASE | 0 | 0 | 0.014528332 | 0.008242342 |
| LMRC | 0 | 0 | 0 | 0.014521706 |
| LMRD | 0 | 0.01056419 | 0.026403359 | 0.020366688 |
| LNUA | 0 | 0 | 0.006119437 | 0.002765388 |
| LNUC | 0.010772497 | 0.059105673 | 0.061710927 | 0.060054705 |
| LNUD | 0.012180667 | 0 | 0.00032183 | 0 |
| LS69_04805 | 0 | 0.000168873 | 0 | 0 |
| LSA | 0 | 0 | 0.016003617 | 0.018061234 |
| LSAA | 0.001970872 | 0.000960133 | 0 | 0 |
| LSAB | 0.012391892 | 0 | 0 | 0 |
| LSAE | 0.032794387 | 0.003109036 | 0.004211371 | 0.005871912 |
| MACA | 0.056321034 | 0.030629556 | 0.002489629 | 0.000109098 |
| MACB | 0.203586981 | 0.068898628 | 0.010340209 | 0.010195623 |
| MAJOR_FACILITATOR_SUPERFAMILY_TRANSPORTER | 0 | 0 | 0.000927158 | 0.00165323 |
| MARA | 0.06528141 | 0.029739159 | 0.002597448 | 0 |
| MDFA | 0.058945977 | 0.038355371 | 0.002435181 | 4.39E-05 |
| MDSB | 0.001619862 | 0.001570245 | 0 | 0 |
| MDTA | 0.059460044 | 0.037776878 | 0.000392142 | 0 |
| MDTB | 0.055503273 | 0.038963168 | 0 | 0 |
| MDTC | 0.055549472 | 0.040089373 | 0 | 0 |
| MDTD | 0.048933742 | 0.030691029 | 0.001811633 | 0 |
| MDTE | 0.032421549 | 0.007012562 | 0.004912437 | 0 |
| MDTF | 0.026902149 | 0.006725337 | 0.004330482 | 0 |
| MDTG | 0.076713173 | 0.047424906 | 0.003183877 | 0.030104708 |
| MDTH | 0.066093594 | 0.045366114 | 0.001936585 | 0 |
| MDTK | 0.10617167 | 0.062438136 | 0.003488983 | 0.000104366 |
| MDTL | 0.091312772 | 0.072446416 | 0.005658229 | 0 |
| MDTM | 0.08173016 | 0.061279066 | 0.004795269 | 0 |
| MDTN | 0.036021223 | 0.016052267 | 0.004154705 | 0 |
| MDTO | 0.026407612 | 0.006731966 | 0.004108557 | 0 |
| MDTP | 0.03906574 | 0.011836873 | 0.004578605 | 0 |
| MDTQ | 0.052637849 | 0.028190446 | 0 | 0 |
| MEFA | 0 | 0.00388766 | 0.002465245 | 0.083326775 |
| MEL | 0 | 0.00219174 | 0.000108378 | 0.083019001 |
| MEXB | 0.01196058 | 0.008976903 | 0.000252295 | 0 |
| MEXE | 0 | 0 | 0.00032129 | 0 |
| MEXF | 0.004106079 | 0.004111885 | 0 | 0 |
| MEXQ | 0.001919015 | 0 | 0 | 0 |
| MEXX | 0 | 0 | 0.003942961 | 0 |
| MFD | 0.070951628 | 0.044557491 | 0 | 0 |
| MGRB | 0.03414961 | 0.013461674 | 0.000655073 | 0 |
| MPRF | 0.00065422 | 0 | 0 | 0 |
| MRDA | 0.067776611 | 0.04547961 | 0 | 0 |
| MSBA | 0.064976621 | 0.03551783 | 0.004791324 | 0.00100454 |
| MSRB | 0.071389057 | 0.044454954 | 0 | 0 |
| MSRC | 0 | 0 | 0 | 0.015648958 |
| MSRE | 0 | 0 | 0 | 0.001648685 |
| MUXB | 0.001837776 | 0.00122249 | 0 | 0 |
| OMP36 | 0 | 0 | 0.000633362 | 0 |
| OMPF | 0 | 0 | 0.008445825 | 0.000102501 |
| OMPR | 0 | 0 | 0.027605403 | 0.010197706 |
| OQXA | 0.048875368 | 0.047877631 | 0 | 5.75E-05 |
| OQXB | 0.028603545 | 0.03113126 | 0 | 0 |
| PATA | 0.08203646 | 0.056715495 | 0.003976717 | 7.84E-05 |
| PATB | 0 | 0 | 0 | 0.03173573 |
| PBP-1A | 0 | 0 | 0 | 0.028228202 |
| PBP2 | 0.025206913 | 0.022939427 | 0 | 0 |
| PENA | 0 | 0 | 0.000564796 | 0.023893882 |
| PMRC | 0.02953243 | 0.008264454 | 0 | 0 |
| PMRE | 0.088596366 | 0.031854017 | 0 | 0 |
| PMRF | 0 | 0.000162469 | 0.002117216 | 0 |
| PORIN_OMPC | 0 | 0 | 0.000239692 | 0 |
| ROBA | 0.078911093 | 0.049484161 | 0 | 0 |
| ROSA | 0.007974283 | 0.004817806 | 0.002113351 | 0 |
| ROSB | 0.067942236 | 0.04701679 | 0.002616327 | 7.45E-05 |
| RPOB2 | 0 | 0 | 0.090366687 | 0.101188065 |
| SAT-4 | 0.010520591 | 0.011001122 | 0.001221763 | 0.000199878 |
| SDIA | 0.028145789 | 0.014630151 | 0.002547375 | 0 |
| SERRATIA_MARCESCENS_OMP1 | 0 | 0 | 0.001164268 | 0 |
| SHV-112 | 0.000560534 | 0.000422018 | 0 | 0 |
| SHV-12 | 0.000996862 | 0.000833916 | 0 | 0 |
| SHV-67 | 0.000322993 | 0.000101324 | 0 | 0 |
| SMEE | 0 | 0.0010449 | 0 | 0 |
| TAEA | 0.019619192 | 0 | 0.00463591 | 0 |
| TET(45) | 0 | 0 | 0 | 0.000216735 |
| TET(W/N/W) | 0 | 0 | 0.001245854 | 0.001111995 |
| TET32 | 0.00310811 | 0.000430824 | 0.009271629 | 0.002273256 |
| TET34 | 0.03943972 | 0.020824424 | 0.001770762 | 0 |
| TET40 | 0.008716296 | 0.016904164 | 0.000628335 | 0.000830772 |
| TET44 | 0.018673648 | 0.000505616 | 0.001532548 | 0.001054042 |
| TETA | 0 | 0 | 0 | 0.013149095 |
| TETB(46) | 0 | 0 | 0 | 0.004100431 |
| TETB(P) | 0 | 0.000133337 | 0.002229611 | 0 |
| TETC | 0.053589878 | 0.031715431 | 0 | 0 |
| TETL | 0 | 0 | 0 | 0.006961896 |
| TETM | 0.010462766 | 0.003227399 | 0.017889357 | 0.120243303 |
| TETO | 0.151610717 | 0.01504104 | 0.001197671 | 0.004011629 |
| TETP | 0 | 0 | 0.001504422 | 0.016162316 |
| TETPA | 0 | 0.000149493 | 0 | 0 |
| TETS | 0 | 0 | 0 | 0.000670397 |
| TETW | 0.109741584 | 0.08773688 | 0.14239212 | 0.153272382 |
| TOLC | 0.075043726 | 0.051256206 | 0.003465363 | 0 |
| TRANSCRIPTIONAL_REGULATORY_PROTEIN_CPXR_CPXR | 0 | 0 | 0.009441275 | 0 |
| TRUNCATED_PUTATIVE_RESPONSE_REGULATOR_ARLR | 0 | 0 | 0.068686485 | 0.059100812 |
| UGD | 0 | 0 | 0.01234484 | 0.018661258 |
| UPPP | 0.32911101 | 0.057537946 | 0 | 0 |
| UPPP_2 | 0.002561175 | 0.000127099 | 0 | 0 |
| VANB | 0 | 0 | 0.000115746 | 0 |
| VANC | 0.001245594 | 0.000520995 | 0.000511435 | 0.001228899 |
| VAND | 0 | 0 | 0 | 0.001193147 |
| VANG | 0.028354644 | 0.002587866 | 0.014822769 | 0.002585032 |
| VANH | 0 | 0 | 0.000422132 | 0 |
| VANR | 0 | 0 | 0.087160218 | 0.056421797 |
| VANRA | 0.16320746 | 0.027473186 | 0 | 0 |
| VANRC | 0.014246287 | 0.001463564 | 0 | 0 |
| VANRD | 0.055246376 | 0.006328652 | 0 | 0 |
| VANRE | 0.001411859 | 0 | 0 | 0 |
| VANRG | 0.101662651 | 0.012557379 | 0 | 0 |
| VANRI | 0.03429257 | 0.006307834 | 0.006711268 | 0.006726486 |
| VANRL | 0.004198906 | 0 | 0 | 0 |
| VANS | 0 | 0 | 0.015438486 | 0.083365081 |
| VANSA | 0.029435851 | 0.003628577 | 0 | 0 |
| VANSC | 0.003313199 | 0.000576278 | 0 | 0 |
| VANSD | 0.024397184 | 0.003384636 | 0 | 0 |
| VANSG | 0.002604348 | 0.000931829 | 0 | 0 |
| VANT | 0 | 0 | 0.000394723 | 0.000973763 |
| VANTC | 0.000430117 | 0.000539715 | 0 | 0 |
| VANTG | 0.001526606 | 0 | 0.000185324 | 0.000252654 |
| VANU | 0.00015396 | 0 | 0.001935272 | 0.001439119 |
| VANUG | 0.002886748 | 0.000317747 | 0 | 0 |
| VANV | 0.046187961 | 0.006143117 | 0 | 0 |
| VANVB | 0 | 0 | 0.007870723 | 0.003471559 |
| VANW | 0 | 0 | 0.000799699 | 0.000294365 |
| VANWG | 0.002547735 | 0 | 0 | 0 |
| VANX | 0 | 0 | 0.00010887 | 0 |
| VANY | 0 | 0 | 0.003012934 | 0.001952933 |
| VANXYC | 0.000607736 | 0.000508396 | 0 | 0 |
| VANXYG | 0.001682042 | 0 | 0 | 0 |
| VANYG1 | 0.004278201 | 0 | 0.000662116 | 0.00154744 |
| VATB | 0.012811661 | 0.000298985 | 0.002157679 | 0.001145525 |
| VATE | 0.002536021 | 0 | 0 | 0 |
| VGAC | 0.0878179 | 0.06450273 | 5.79E-05 | 0 |
| YBJZ | 0.00250395 | 0.000301389 | 0 | 0 |
| YFBF | 0.019421543 | 0.019703062 | 0 | 0 |
| YFBG | 0.038997335 | 0.045019227 | 0 | 0 |
| YOJI | 0.061450254 | 0.04190502 | 0.002106703 | 0 |
